# Certifiably optimal rotation and pose estimation based on the Cayley map

**DOI:** 10.1177/02783649241269337

**Published:** 2024-09-25

**Authors:** Timothy D. Barfoot, Connor Holmes, Frederike Dümbgen

**Affiliations:** Robotics Institute, 7938 University of Toronto, Toronto, ON, Canada

**Keywords:** Rotation estimation, pose estimation, quadratically constrained quadratic program, semi-definite program, Lagrangian duality, Cayley map

## Abstract

We present novel, convex relaxations for rotation and pose estimation problems that can *a posteriori* guarantee global optimality for practical measurement noise levels. Some such relaxations exist in the literature for specific problem setups that assume the matrix von Mises-Fisher distribution (a.k.a., matrix Langevin distribution or chordal distance) for isotropic rotational uncertainty. However, another common way to represent uncertainty for rotations and poses is to define anisotropic noise in the associated Lie algebra. Starting from a noise model based on the Cayley map, we define our estimation problems, convert them to Quadratically Constrained Quadratic Programs (QCQPs), then relax them to Semidefinite Programs (SDPs), which can be solved using standard interior-point optimization methods; global optimality follows from Lagrangian strong duality. We first show how to carry out basic rotation and pose averaging. We then turn to the more complex problem of trajectory estimation, which involves many pose variables with both individual and inter-pose measurements (or motion priors). Our contribution is to formulate SDP relaxations for all these problems based on the Cayley map (including the identification of redundant constraints) and to show them working in practical settings. We hope our results can add to the catalogue of useful estimation problems whose solutions can be *a posteriori* guaranteed to be globally optimal.

## 1. Introduction

*State estimation* is concerned with fusing several noisy measurements (and possibly a prior model) into a less noisy estimate of the state (e.g., position, velocity, orientation) of a vehicle, robot, or other object of interest. Real-world state estimation problems often involve measurement functions and motion models that are nonlinear with respect to the state. Alternatively, the state itself may not be an element of a vector space, such as the rotation of a rigid body. These challenging aspects typically mean that when we set up our estimator as an optimization, it is a *nonconvex* problem; the cost function, the feasible set, or both are not convex. Nonconvex optimization problems are in general much harder to solve than convex ones because they can have local minima and common gradient-based optimizers can easily become trapped therein.

For example, we might have a generic nonlinear least-squares problem such as

(1) $$\underset{x}{\text{min}}\overset{M}{\underset{m=1}{\sum }}{\left(y_{m}-g\left(x\right)\right)}^{T}\left(y_{m}-g\left(x\right)\right),$$

where **x** is the unknown state, **y**_*m*_ are noisy measurements, and **g**(⋅) is a measurement function. If **g**(**x**) is linear in **x**, then this problem is convex, but otherwise it often is not. Using gradient descent or Gauss-Newton to solve this problem means we usually require a good initial guess for **x** to arrive at the global minimum. What if such an initial guess is not available? Could we solve a problem such as this one globally without such a guess? It turns out the answer may be yes, depending on the specific problem to be solved.

There has been quite a bit of work in robotics and computer vision aimed at the idea of solving estimation problems globally. Most of these works employ sophisticated tools from the optimization literature to achieve this. In particular, *Lagrangian duality* is used to derive *convex relaxations*, which can be solved globally. Boyd and Vandenberghe (2004, §5) provide the necessary background on duality theory. We will be following a common pathway where we first convert our nonconvex optimization problem into another nonconvex form called a Quadratically Constrained Quadratic Program (QCQP); from here we relax this to a (convex) Semi-definite Program (SDP), amenable to off-the-shelf solvers (e.g., the interior-point-based SDP solver in mosek (ApS, 2019)). This last step is known as *Shor’s relaxation* (Shor, 1987). Theoretically, SDPs admit polynomial-time solutions because they are convex; in practice, modern SDP problems with a few thousand variables can be solved in reasonable time but struggle to scale up beyond this. Our contribution in this paper is to show that we can solve a set of estimation problems involving rotations and poses globally, using these convex relaxation tools; the novelty lies in the fact that our particular problems (formulated using the Cayley map) have not been examined in this globally optimal framework before.

As mentioned, the convex relaxation procedure we employ has been well known in the optimization community for some time. In particular, it has been used for *polynomial optimization* (Parrilo, 2003) and in various *combinatorial optimization* problems, such as *quadratic assignment* (Nesterov et al., 2000) and *max-cut graph partitioning* (Anjos and Wolkowicz, 2002). To the authors’ knowledge, the first use of SDP relaxations in the robotics community was by Liu et al. (2012) for *planar Simultaneous Localization and Mapping (SLAM)*, though their application in computer vision (Kahl and Henrion, 2005) and signal processing (Krislock and Wolkowicz, 2010; Luo et al., 2010) occurred earlier. More generally, Cifuentes et al. (2022) provides a nice overview of some common problems in computer vision and robotics where these tools have been applied before, as well as providing a rationale for why they are so effective. One of the most commonly investigated relaxations in the robotics and vision communities is *rotation synchronization*^1^ (Bandeira et al., 2017; Dellaert et al., 2020; Eriksson et al., 2018, 2021); here several rotations are linked through noisy relative rotation measurements. Rotation synchronization turns out to be the nucleus of several of the other problems under study including *pose-graph optimization* (Briales and Gonzalez-Jimenez, 2017a; Carlone and Calafiore, 2018; Carlone et al., 2016; Rosen et al., 2019; Tian et al., 2021), *point-set registration* (Chaudhury et al., 2015; Iglesias et al., 2020; Yang et al., 2021), *calibration* (Giamou et al., 2019; Wise et al., 2020), *mutual localization* (Wang et al., 2022b), and *landmark-based SLAM* (Holmes and Barfoot, 2023). Rotation synchronization and its cousins have been shown to admit fast solutions through low-rank factorizations (Rosen et al., 2019). More recently, other measurement models such as *range sensing* have also been incorporated into globally certifiable problems (Dümbgen et al., 2023; Papalia et al., 2023).

It is worth also mentioning the works of Forbes and De Ruiter (2015); Horowitz et al. (2014) on globally optimal pointcloud alignment, which also employ convex relaxation, but the route to the SDP is not Shor’s relaxation; instead, Lie group optimization variables are relaxed to live in a convex set. On the surface, this approach is not applicable to the problems considered herein; for example, our cost functions are not always initially convex.

A common thread that ties most of the existing literature together is that the *chordal distance* is used to construct the terms of the cost function that involve rotation variables. Viewed through a probabilistic lens, the chordal distance is related to the matrix Langevin or matrix von Mises-Fisher distribution, whose density can be written in the form

(2) $$p\left(C\right)\propto \exp\left(-\frac{1}{2}\sigma^{-1}\;\text{tr}\left(\left(C-\bar{C}\right){\left(C-\bar{C}\right)}^{T}\right)\right),$$

where **C** is a rotation matrix, $\bar{C}$ is the mode, and *σ* > 0 is a scalar concentration parameter; this distribution is isotropic, which is one limitation we aim to overcome in this work. This is not the only way to represent rotational uncertainty. Another common way is to use exponential coordinates (e.g., Barfoot and Furgale (2014); Long et al. (2013)), where a rotational distribution can have a density of the form

(3) $$p\left(C\right)\propto \exp\left(-\frac{1}{2}\ln{\left(C{\bar{C}}^{T}\right)}^{\vee^{T}}\Sigma^{-1}\ln{\left(C{\bar{C}}^{T}\right)}^{\vee }\right),$$

where **C** is a member of the matrix Lie group *SO*(3), $\bar{C}$ is also a member of *SO*(3) and represents the mean, and **Σ** is an anisotropic matrix covariance. We also have exp(⋅) as the matrix exponential, ln(⋅) the matrix logarithm, and ∨ a Lie algebra operator detailed a bit later in the paper. Here we are essentially defining a Gaussian distribution in the vector space of the Lie algebra associated with *SO*(3) and then mapping the uncertainty to the Lie group through the matrix exponential. This allows for anisotropic distributions and the same approach can be easily extended to any matrix Lie group, such as the special Euclidean group *SE*(3) that represents poses (see, e.g., Barfoot and Furgale (2014); Long et al. (2013)). Our aim in this paper is to present some novel convex relaxations where rotational uncertainty is defined closer to this exponential coordinate model; to achieve this, we use the Cayley map, which is very close to the exponential map for small-to-moderate rotational uncertainty. Our Cayley distributions will have the form

(4) $$p\left(C\right)\propto \text{cay}\left(-\frac{1}{2}{\text{cay}}^{-1}{\left(C{\bar{C}}^{T}\right)}^{\vee^{T}}\Sigma^{-1}{\text{cay}}^{-1}{\left(C{\bar{C}}^{T}\right)}^{\vee }\right),$$

where cay(⋅) is the Cayley map. This also allows us to define our optimization problems directly on *SE*(3) rather than $SO\left(3\right)\times R^{3}$ when poses are involved.

To our knowledge, the examination of global optimality for state estimation problems where rotational (and pose) uncertainty is defined in this way has not be explored previously in the literature. Our novel contribution is therefore a family of specific convex relaxations of rotation and pose estimation problems formulated using the Cayley map (including redundant constraints needed to make them work in practice); this is important as it opens the door to providing certification for a broad class of state estimation problems used in practice.

This paper is organized as follows. In Section 2, we review the relevant mathematical background including Lie groups, the Cayley map, and the convex relaxation procedure that we will employ. Section 3 presents the basic problems of averaging a number of noisy rotation or pose measurements. In Section 4, we expand the method to include discrete-time trajectory estimation of several poses based on individual and inter-pose measurements. Section 5 expands this to include so-called continuous-time trajectory estimation where we have a smoothing assumption on the trajectory and estimate both pose and twist at each state. In each of Sections 3 to 5, we provide experimental results that demonstrate the viability of our convex relaxations to find globally optimal solutions. Section 6 concludes the paper. Appendix A discusses the similarity between distributions defined using the exponential and Cayley maps while Appendix B presents the baseline local solvers to which we compare our new global estimates.

## 2. Mathematical background

We begin by reviewing the relevant background concepts for the paper including Lie groups, the Cayley map, and convex relaxations of nonconvex optimization problems via Lagrangian duality.

### 2.1. Lie groups for rotations and poses

The *special orthogonal group*, representing rotations, is the set of valid rotation matrices:

(5) $$SO\left(3\right)=\left\{C\in R^{3\times 3}\;|CC^{T}=I,\text{det}\left(C\right)=1\right\},$$

where **I** is the identity matrix. It is common to map a vector, $\phi \in R^{3}$ , to a rotation matrix, **C**, through the matrix exponential,

(6) $$C\left(\phi \right)=\exp\left(\phi^{\wedge }\right),$$

where (⋅)^∧^ is the skew-symmetric operator,

(7) $$\phi^{\wedge }={\left[\begin{array}{c} \phi_{1}\\ \phi_{2}\\ \phi_{3} \end{array}\right]}^{\wedge }=\left[\begin{array}{ccc} 0 & -\phi_{3} & \phi_{2}\\ \phi_{3} & 0 & -\phi_{1}\\ -\phi_{2} & \phi_{1} & 0 \end{array}\right],$$

and $\phi =\varphi a\in R^{3}$ , the product of the angle and unit axis of rotation. The mapping is surjective-only, meaning every **C** can be produced by many different values for ***ϕ***.

The *special Euclidean group*, representing poses (i.e., translation and rotation), is the set of valid transformation matrices:

(8) $$SE\left(3\right)=\left\{T=\left[\begin{array}{cc} C & r\\ \;\;0^{T} & 1 \end{array}\right]\in R^{4\times 4}\;|C\in SO\left(3\right),\;r\in R^{3}\right\}.$$

It is again common to map a vector, $\xi \in R^{6}$ , to a transformation matrix, **T** ∈ *SE*(3), through the matrix exponential,

(9) $$T\left(\xi \right)=\exp\left(\xi^{\wedge }\right),$$

where we have overloaded the ∧ operator as

(10) $$\xi^{\wedge }={\left[\begin{array}{c} \rho \\ \phi \end{array}\right]}^{\wedge }=\left[\begin{array}{cc} \phi^{\wedge } & \rho \\ 0^{T} & 0 \end{array}\right].$$

Notationally, we will use ∨ to mean the inverse operation of ∧. As is common practice (Barfoot, 2024), we have broken the pose vector, ***ξ***, into a translational component, ***ρ***, and a rotational component, ***ϕ***. The mapping is also surjective-only, meaning every **T** can be produced by many different values for ***ξ***.

Finally, the *adjoint* of a pose is given by

(11) $$T\left(\xi \right)=\text{Ad}\left(T\right)=\left[\begin{array}{cc} C & r^{\wedge }C\\ 0 & C \end{array}\right],$$

which is now 6 × 6. We will refer to the set of adjoints as Ad(*SE*(3)). We can map a vector, $\xi \in R^{6}$ , to an adjoint transformation matrix again through the matrix exponential map:

(12) $$T\left(\xi \right)=\exp\left(\xi^{⋏}\right),$$

where

(13) $$\xi^{⋏}={\left[\begin{array}{c} \rho \\ \phi \end{array}\right]}^{⋏}=\left[\begin{array}{cc} \phi^{\wedge } & \rho^{\wedge }\\ 0 & \phi^{\wedge } \end{array}\right].$$

Notationally, we will use ⋎ to mean the inverse operation of ⋏. The mapping is again surjective-only, meaning every T can be produced by many different values for ***ξ***.

### 2.2. Cayley map

While the exponential map is the canonical way to map from a Lie algebra (vector space) to a Lie group, it is not the only possibility. There are in fact infinitely many such vectorial mappings for *SO*(3) (Bauchau and Trainelli, 2003), *SE*(3) (Barfoot et al., 2022), and Ad(*SE*(3)) (Bauchau, 2011; Bauchau and Choi, 2003).

In particular, it is well known that for the Cayley-Gibbs-Rodrigues parameterization of rotation we can write the rotation matrix in terms of the Cayley map, $\text{cay}\left(A\right)={\left(I-\frac{1}{2}A\right)}^{-1}\left(I+\frac{1}{2}A\right)$ , according to Bauchau and Trainelli (2003); Borri et al. (2000):

(14a) $$\begin{array}{ll} C\left(\phi \right) & =\text{cay}\left(\phi^{\wedge }\right)={\left(I-\frac{1}{2}\phi^{\wedge }\right)}^{-1}\left(I+\frac{1}{2}\phi^{\wedge }\right), \end{array}$$

(14b) $$\begin{array}{ll} \phi & ={\text{cay}}^{-1}{\left(C\right)}^{\vee }={\left(2\left(C-I\right){\left(C+I\right)}^{-1}\right)}^{\vee }, \end{array}$$

for some $\phi =2\tan\frac{\varphi }{2}a\in R^{3}$ with *φ* the rotation angle and **a** the unit axis. Borri et al. (2000) and later Selig (2007) demonstrated that the Cayley map can also be used to map pose vectors to *SE*(3) according to

(15a) $$\begin{array}{ll} T\left(\xi \right) & =\text{cay}\left(\xi^{\wedge }\right)={\left(I-\frac{1}{2}\xi^{\wedge }\right)}^{-1}\left(I+\frac{1}{2}\xi^{\wedge }\right), \end{array}$$

(15b) $$\begin{array}{ll} \xi & ={\text{cay}}^{-1}{\left(T\right)}^{\vee }={\left(2\left(T-I\right){\left(T+I\right)}^{-1}\right)}^{\vee }, \end{array}$$

for some $\xi \in R^{6}$ . Although we will not need it, the Cayley map can be used to map pose vectors to Ad(*SE*(3)) according to

(16a) $$\begin{array}{ll} T\left(\xi \right) & =\text{cay}\left(\xi^{⋏}\right)={\left(I-\frac{1}{2}\xi^{⋏}\right)}^{-1}\left(I+\frac{1}{2}\xi^{⋏}\right), \end{array}$$

(16b) $$\begin{array}{ll} \xi & ={\text{cay}}^{-1}{\left(T\right)}^{⋎}={\left(2\left(T-I\right){\left(T+I\right)}^{-1}\right)}^{⋎}.\; \end{array}$$

However, Selig (2007) demonstrates that starting from the same ***ξ*** and applying (15a), (15b), (16a) and (16b) does not result in an equivalent transformation, that is, T(ξ)≠Ad(T(ξ)) ; the commutative property for adjoints does not hold. Nevertheless, we shall not require this property here.

**Figure 1.**
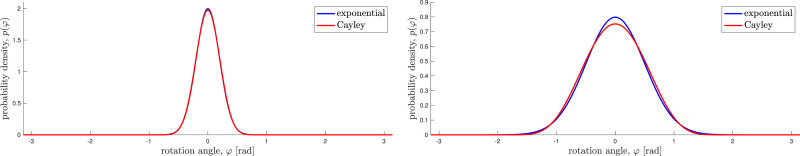
Comparison of uncertainty on rotation angle, *φ*, for the exponential and Cayley maps, where the variances have been approximately matched (see Appendix A for further discussion of how this was done). (left) Standard deviation of rotational uncertainty is *σ* = 0.2 [rad]. (right) *σ* = 0.5 [rad]. The match is good in both cases with more divergence as rotational uncertainty increases.

Figure 1 provides examples comparing two rotational distributions derived from the exponential and Cayley maps that have approximately the same variance; we can see that even with quite large rotational uncertainty they match quite closely. Appendix A provides some further discussion on how closely these distributions can be made to match. The key idea of the paper will be to replace instances of the exponential map with the Cayley map, which we will see is more amenable to producing polynomial optimization problems, a key prerequisite to our route to global optimality.

The Cayley map has been used in the past for rotation, pose, and trajectory estimation (Alismail et al., 2014; Barfoot et al., 2022; Junkins et al., 2011; Majji et al., 2011; Mortari et al., 2007; Qian et al., 2020; Wong et al., 2018; Wong and Majji, 2016), typically to parameterize rotations or poses thereby creating a simpler unconstrained quadratic optimization problem. The drawback of these approaches is that they are still subject to singularities and local minima. The Cayley map has also been used to achieve global optimality in the perspective-*n*-point (PnP) problem (Nakano, 2015; Wang et al., 2022a); we take a quite different approach, however, through the use of convex relaxations. Additionally, the Cayley map has found application in parametrizing lines in structure from motion, as an unconstrained alternative to parametrizations such as Plücker coordinates (Zhang and Koch, 2014).

The Cayley map has also been employed in areas of robotics other than estimation. In Kobilarov (2014); Kobilarov and Marsden (2011), for example, the authors suggest using the Cayley map for rotation parametrization in the context of optimal control of mechanical systems on Lie groups. The authors observe that, compared with the exponential map, the Cayley map is computationally more efficient because of its simpler structure, in particular as it circumvents trigonometric functions. It is also noted that the Cayley map has no singularities in its gradients, which is of advantage for the numerical stability of commonly used solvers (Kobilarov and Marsden, 2011). In Solo and Wang (2019), the Cayley map is employed for simulating stochastic differential equations that evolve on Stiefel manifolds, which is subsequently used in Wang and Solo (2020) for a novel particle filter variant. It is possible that our global optimality approach to using the Cayley map could be employed within some of these applications, but we leave this investigation for future work.

### 2.3. Convex relaxations

We next summarize the key optimization tools that we will use. Boyd and Vandenberghe (2004) provide the appropriate background. Suppose that we have a nonconvex optimization problem of the form

(17) $$\begin{array}{ccc} \min & f\left(z\right) & \\ w.r.t. & z & \\ s.t. & g_{i}\left(z\right)=0\;(\forall i). & \end{array}$$

We attempt to introduce appropriate nonlinear substitution variables, **x**, to replace **z** so that both the objective and the constraints can be written in a standard, homogeneous, quadratic form:

(18) $$\begin{array}{ccc} \min & x^{T}Qx & \\ w.r.t. & x & \\ s.t. & x^{T}A_{0}x=1 & \\ & x^{T}A_{i}x=0\;\left(\forall i\neq 0\right). & \end{array}$$

This problem is a Quadratically Constrained Quadratic Program (QCQP), which is still nonconvex and typically of higher dimension than the original problem, but possesses more exploitable structure. Next, by defining **X** = **xx**^*T*^, we rewrite this problem exactly as

(19) $$\begin{array}{ccc} \min & \text{tr}\left(QX\right) & \\ w.r.t. & X & \\ s.t. & X⪰0 & \\ & \text{rank}\left(X\right)=1 & \\ & \text{tr}\left(A_{0}X\right)=1 & \\ & \text{tr}\left(A_{i}X\right)=0\;(\forall i\neq 0). & \end{array}$$

Finally, if we drop the rank(**X**) = 1 constraint, we have a *convex relaxation* of the problem in the form of a Semidefinite Program (SDP):

(20) $$\begin{array}{ccc} \min & \text{tr}\left(QX\right) & \\ w.r.t. & X & \\ s.t. & X⪰0 & \\ & \text{tr}\left(A_{0}X\right)=1 & \\ & \text{tr}\left(A_{i}X\right)=0\;(\forall i\neq 0). & \end{array}$$

This is known as *Shor’s relaxation* (Shor, 1987). If the solution to this problem happens to result in rank(**X**) = 1, then we have an a posteriori^2^ guarantee that we also have a global solution to the original problem, **x**. Since SDPs are convex problems, we can attempt to use standard solvers, such as interior-point methods, to solve them numerically. While there are tractability issues to be addressed to scale up to very large problem instances, we will see that for the problems in this paper, this approach is viable for nontrivial problem sizes.

Unfortunately, for most problems in this paper, Shor’s relaxation is not *tight* out of the box. In this paper, tightness means rank(**X**) = 1 (and therefore that the optimal cost matches that of the original problem). However, there is still a way forward. We can attempt to introduce additional so-called *redundant constraints* to tighten up the relaxation. These constraints do not affect the feasible set of the original optimization problem, but they do reduce the feasible set of the SDP in order to tighten it.

The technique of adding redundant constraints to improve the tightness of a given SDP relaxation has been known for some time in the optimization literature (Anstreicher and Wolkowicz, 2000; Nesterov et al., 2000). More recently, there have been several cases in which redundant constraints have been used in the robotics (Yang et al., 2021; Yang and Carlone, 2023; Giamou et al., 2019; Wang et al., 2022b) and machine vision (Briales et al., 2018; Briales and Gonzalez-Jimenez, 2017b; Garcia-Salguero et al., 2022; Kezurer et al., 2015) literature. With redundant constraints, our problem becomes

(21) $$\begin{array}{ccc} \min & \text{tr}\left(QX\right) & \\ w.r.t. & X & \\ s.t. & X⪰0 & \\ & \text{tr}\left(A_{0}X\right)=1 & \\ & \text{tr}\left(A_{i}X\right)=0\;\left(\forall i\neq 0\right) & \\ \left(redundant\right) & \text{tr}\left(B_{j}X\right)=0\;(\forall j\neq 0), & \end{array}$$

where the **B**_*j*_ encapsulate these additional redundant constraints. In theory, Lasserre (2001) tells us how to tighten our SDP, if possible, by adding a progression of variables and constraints, but adding too many constraints can be computationally expensive and in practice not necessary for tightness.^3^ On the other hand, devising a sufficient set of constraints can be challenging by trial and error. In our concurrent work, we have been developing a tool to automatically find such constraints (Duembgen et al., 2023), which we used to help identify some of the constraints reported in this paper. In all of the pose estimation problems to follow, we do require redundant constraints and we will be explicit in enumerating ones that in practice result in tight SDP relaxations of our problems. This could be viewed as the core contribution of the paper.

## 3. Averaging

We will build up our optimization problems gradually starting with simply ‘averaging’ several noisy estimates of rotation or pose.

### 3.1. Rotation averaging

In order to average *M* rotations, we could set up an optimization problem as

(22) $$\begin{array}{ccc} \min & \overset{M}{\underset{m=1}{\sum }}\ln{\left(C{\tilde{C}}_{m}^{T}\right)}^{\vee^{T}}W_{m}\;\ln{\left(C{\tilde{C}}_{m}^{T}\right)}^{\vee } & \\ w.r.t. & C & \\ s.t. & C\in SO\left(3\right), & \end{array}$$

where ${\tilde{C}}_{m}\in SO\left(3\right)$ are the noisy rotations to be averaged and **W**_*m*_ is a matrix weight. This type of cost function is used frequently in rotational estimation problems (Barfoot, 2024) and can represent a maximum-likelihood problem when the generative model for the noisy measurements is of the form

(23) $${\tilde{C}}_{m}=\exp\left(\phi_{m}^{\wedge }\right)C,\;\phi_{m}∼N(0,W_{m}^{-1}).$$

Alternatively, we can view our cost as the negative log-likelihood of the joint distribution of the measurements if each obeys (3). The trouble is that the matrix exponential and logarithm are difficult expressions to manipulate into the QCQP form we seek.

This is where the main insight of the paper comes in. We can substitute the Cayley map for the exponential map without too much effect on the stated problem (see Figure 1). With this substitution, our generative model for noisy rotations becomes

(24) $${\tilde{C}}_{m}=\text{cay}\left(\phi_{m}^{\wedge }\right)C,\;\phi_{m}∼N(0,W_{m}^{-1}),$$

and so our optimization problem can be restated as

(25) $$\begin{array}{cc} \min & \overset{M}{\underset{m=1}{\sum }}{\text{cay}}^{-1}{\left(C{\tilde{C}}_{m}^{T}\right)}^{\vee^{T}}W_{m}\;{\text{cay}}^{-1}{\left(C{\tilde{C}}_{m}^{T}\right)}^{\vee }\\ w.r.t. & C\\ s.t. & C\in SO\left(3\right). \end{array}$$

Now our cost represents the negative log-likelihood of the joint distribution of the measurements, assuming each obeys (4). Turning this into a QCQP is then fairly easy:

(26) $$\begin{array}{ccc} \min & \overset{M}{\underset{m=1}{\sum }}\phi_{m}^{T}W_{m}\phi_{m} & \\ w.r.t. & c_{1},c_{2},c_{3},\phi_{m}\;\left(\forall m\right) & \\ s.t. & c_{i}^{T}c_{j}=\delta_{ij}\;\left(\forall i,j\right) & \\ & \left(I-\frac{1}{2}\phi_{m}^{\wedge }\right)c_{i}=\left(I+\frac{1}{2}\phi_{m}^{\wedge }\right){\tilde{c}}_{m,i}\;(\forall i,m), & \end{array}$$

where *δ*_*ij*_ is the Kronecker delta and

(27) $$C=\left[\begin{array}{ccc} c_{1} & c_{2} & c_{3} \end{array}\right],\;{\tilde{C}}_{m}=\left[\begin{array}{ccc} {\tilde{c}}_{m,1} & {\tilde{c}}_{m,2} & {\tilde{c}}_{m,3} \end{array}\right].$$

We have essentially introduced variables, ***ϕ***_*m*_, for the residual errors of each term in the cost and used these to connect **C** to each ${\tilde{C}}_{m}$ through the Cayley map; by bringing the inverse factor of the Cayley map to the other side, this becomes a quadratic constraint. Thus we have both a quadratic cost and quadratic constraints and hence a QCQP. The dimension of the problem is now higher since we must now optimize over **C** and all the ***ϕ***_*m*_; however, we can follow the approach of Section 2.3 to produce a SDP relaxation of the problem. Note, we have quietly dropped the det(**C**) = 1 constraint on the rotation and will simply check it at the end^5^; our optimization then only guarantees **C** ∈ *O*(3) not *SO*(3). We leave the details of manipulating (26) into the standard form of (20) to the reader. We did not find any redundant constraints were necessary to tighten this relaxation; the SDP solution produced remains rank 1 in practice for reasonably high amounts of noise.

**Figure 2.**
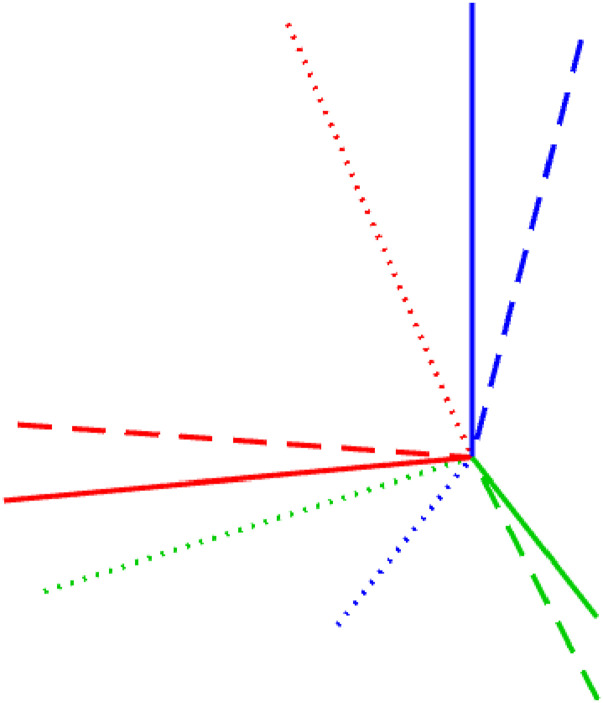
*Rotation Averaging:* An example of noisy rotation averaging where the randomly initialized local solver (dotted) becomes trapped in a poor local minimum while the global solver (dashed) finds the correct global solution, which is closer to the groundtruth rotation (solid).

**Figure 3.**
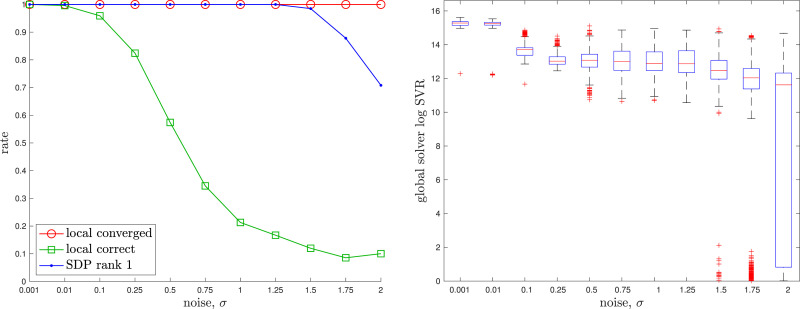
*Rotation Averaging:* A quantitative evaluation of the tightness of the rotation averaging problem with increasing measurement noise level, *σ*. At each noise level, we conducted 1000 trials of averaging 10 noisy rotations. (left) We see that the local solver (randomly initialized) finds the global minimum with decreasing frequency (green) as the measurement noise is increased, while the SDP solver (blue) successfully produces rank-1 solutions (we consider log SVR of at least 5 to be rank 1) to much higher noise levels. For completeness, we also show how frequently the local solver converges to any minimum (red). (right) Boxplots of the log SVR of the SDP solution show that the global solution remains highly rank 1 over a wide range of measurement noise values.

The details of a baseline local solver can be found in the appendix. For the global (SDP) solver we used cvx in Matlab with mosek (ApS, 2019). The solution costs of the global and local solvers agree to high precision^6^ if a good initial guess is given to the local solver. Figure 2 provides an example where the local solver converges from a poor initial guess to a local minimum, while the global solver finds the optimal solution near the groundtruth. Figure 3 provides a quantitative study of the tightness of the SDP solution with increasing measurement noise; we selected the measurement covariance as $W_{m}^{-1}=\sigma^{2}I$ , with *σ* increasing. To gauge numerically whether the SDP solution, **X**, is rank 1, we define the *logarithmic Singular Value Ratio (SVR)* as the base-10 logarithm of the ratio of the largest to second-largest singular values of **X**; we consider a log SVR of at least 5 to represent rank 1. We see there is a large range for the noise over which the local solver can become trapped in a local minimum while the global solver remains rank 1. With *M* = 10 rotations to be averaged, the local solver took on average 0.0012*s* while the SDP solver took on average 0.3486*s*.

### 3.2. Pose averaging

Pose averaging follows a very similar approach to the previous section.^4^ An optimization problem based on the Cayley map can be stated as

(28) $$\begin{array}{ccc} \min & \overset{M}{\underset{m=1}{\sum }}{\text{cay}}^{-1}{\left(T{\tilde{T}}_{m}^{-1}\right)}^{\vee^{T}}W_{m}\;{\text{cay}}^{-1}{\left(T{\tilde{T}}_{m}^{-1}\right)}^{\vee } & \\ w.r.t. & T & \\ s.t. & T\in SE(3), & \end{array}$$

where ${\tilde{T}}_{m}$ are noisy pose measurements with matrix weights, **W**_*m*_.

We convert the residual pose errors, $\xi_{m}={\text{cay}}^{-1}{\left(T{\tilde{T}}_{m}^{-1}\right)}^{\vee }$ , to variables and now our optimization can be stated as

(29) $$\begin{array}{ccc} \min & \overset{M}{\underset{m=1}{\sum }}\xi_{m}^{T}W_{m}\xi_{m} & \\ w.r.t. & T,\xi_{m}\;\left(\forall m\right) & \\ s.t. & C^{T}C=I & \\ & \left(I-\frac{1}{2}\xi_{m}^{\wedge }\right)T=\left(I+\frac{1}{2}\xi_{m}^{\wedge }\right){\tilde{T}}_{m}\;(\forall m), & \end{array}$$

where we have again dropped the det(**C**) = 1 constraint. Since the bottom row of a transformation matrix is constant, we can parameterize it in the following way

(30a) $$\begin{array}{ll} T & =\left[\begin{array}{cc} C & r\\ 0^{T} & 1 \end{array}\right]=\left[\begin{array}{cccc} c_{1} & c_{2} & c_{3} & r\\ 0 & 0 & 0 & 1 \end{array}\right], \end{array}$$

(30b) $$\begin{array}{ll} {\tilde{T}}_{m} & =\left[\begin{array}{cc} {\tilde{C}}_{m} & r_{m}\\ 0^{T} & 1 \end{array}\right]=\left[\begin{array}{cccc} {\tilde{c}}_{m,1} & {\tilde{c}}_{m,2} & {\tilde{c}}_{m,2} & {\tilde{r}}_{m}\\ 0 & 0 & 0 & 1 \end{array}\right], \end{array}$$

(30c) $$\begin{array}{ll} \xi_{m} & =\left[\begin{array}{c} \rho_{m}\\ \phi_{m} \end{array}\right], \end{array}$$

and then rewrite the optimization problem using the reduced set of variables as

(31) $$\begin{array}{ccc} \min & \overset{M}{\underset{m=1}{\sum }}\xi_{m}^{T}W_{m}\xi_{m} & \\ w.r.t. & c_{i},r,\rho_{m},\phi_{m}\;\left(\forall i,m\right) & \\ s.t. & c_{i}^{T}c_{j}=\delta_{ij}\;\left(\forall i,j\right) & \\ & \left(I-\frac{1}{2}\phi_{m}^{\wedge }\right)c_{i}=\left(I+\frac{1}{2}\phi_{m}^{\wedge }\right){\tilde{c}}_{m,i}\;\left(\forall i,m\right) & \\ & \left(I-\frac{1}{2}\phi_{m}^{\wedge }\right)r=\left(I+\frac{1}{2}\phi_{m}^{\wedge }\right){\tilde{r}}_{m}+\rho_{m}\;(\forall m). & \end{array}$$

This is now a QCQP, but unfortunately when we relax to a SDP, it is not always tight even for low-noise levels. We found that introducing specific redundant constraints for each *m* tightens the problem nicely for practical noise levels. One such useful constraint can be found by premultiplying the last constraint in (31) by **r**^*T*^ whereupon

(32) $$r^{T}r-\frac{1}{2}\underset{0}{\underbrace{r^{T}\phi_{m}^{\wedge }r}}=r^{T}{\tilde{r}}_{m}-\frac{1}{2}r^{T}{\tilde{r}}_{m}^{\wedge }\phi_{m}+r^{T}\rho_{m.}$$

The key is that the second cubic term vanishes, leaving a new quadratic constraint that it is not simply a trivial linear combination of the existing constraints (Yang and Carlone, 2023). However, this constraint is redundant because it does not restrict the original feasible set at all. In the lifted SDP space it serves to restrict the feasible set and ultimately tighten the relaxation. Another useful redundant constraint can be formed by combining the last two of (31); the second last can be written as

(33) $$\frac{1}{2}{\left(c_{i}+{\tilde{c}}_{m,i}\right)}^{T}\phi_{m}^{\wedge }=-{\left(c_{i}-{\tilde{c}}_{m,i}\right)}^{T},$$

while the last can be premultiplied by ${\left(c_{i}+{\tilde{c}}_{m,i}\right)}^{T}$ and written as

(34) $$\begin{array}{l} {\left(c_{i}+{\tilde{c}}_{m,i}\right)}^{T}\left(r-{\tilde{r}}_{m}\right)=\underset{-{\left(c_{i}-{\tilde{c}}_{m,i}\right)}^{T}}{\underbrace{\frac{1}{2}{\left(c_{i}+{\tilde{c}}_{m,i}\right)}^{T}\phi_{m}^{\wedge }}}\left(r+{\tilde{r}}_{m}\right)\\ +{\left(c_{i}+{\tilde{c}}_{m,i}\right)}^{T}\rho_{m}. \end{array}$$

After performing the indicated substitution, this becomes

(35) $$\frac{1}{2}{\left(c_{i}+{\tilde{c}}_{m,i}\right)}^{T}\rho_{m}=c_{i}^{T}r-{\tilde{c}}_{m,i}^{T}{\tilde{r}}_{m},$$

which is once again a quadratic constraint.

Summarizing, the following QCQP offers a reasonably tight SDP relaxation in practice:

(36) $$\begin{array}{ccc} \min & \overset{M}{\underset{m=1}{\sum }}\xi_{m}^{T}W_{m}\xi_{m} & \\ w.r.t. & c_{i},r,\rho_{m},\phi_{m}\;\left(\forall i,m\right) & \\ s.t. & c_{i}^{T}c_{j}=\delta_{ij}\;\left(\forall i,j\right) & \\ & \left(I-\frac{1}{2}\phi_{m}^{\wedge }\right)c_{i}=\left(I-\frac{1}{2}\phi_{m}^{\wedge }\right){\tilde{c}}_{m,i}\;\left(\forall i,m\right) & \\ & \left(I-\frac{1}{2}\phi_{m}^{\wedge }\right)r=\left(I+\frac{1}{2}\phi_{m}^{\wedge }\right){\tilde{r}}_{m}+\rho_{m}\;\left(\forall m\right) & \\ \left(red.\right) & \frac{1}{2}{\left(c_{i}+{\tilde{c}}_{m,i}\right)}^{T}\rho_{m}=c_{i}^{T}r-{\tilde{c}}_{m,i}^{T}{\tilde{r}}_{m}\;\left(\forall i,m\right) & \\ & r^{T}r=r^{T}{\tilde{r}}_{m}-\frac{1}{2}r^{T}{\tilde{r}}_{m}^{\wedge }\phi_{m}+r^{T}\rho_{m}\;(\forall m). & \end{array}$$

We leave it to the reader to manipulate this into the standard form of (21).

**Figure 4.**
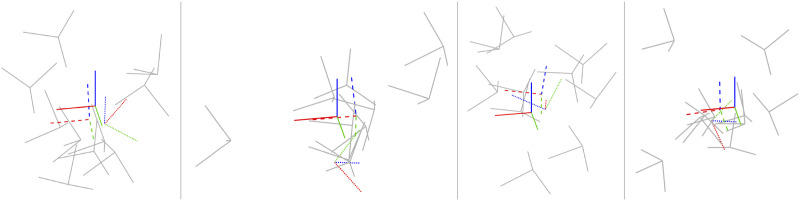
*Pose Averaging:* Four examples of noisy pose averaging where the randomly initialized local solver (dotted) becomes trapped in a poor local minimum while the global solver (dashed) finds the correct solution, which is closer to the groundtruth pose (solid). The noisy pose measurements being averaged are shown in grey.

**Figure 5.**
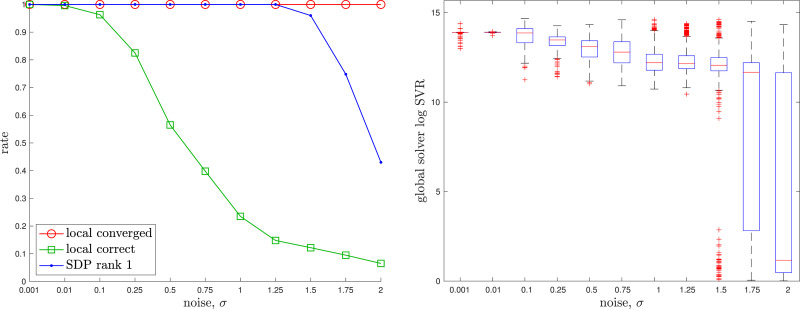
*Pose Averaging:* A quantitative evaluation of the tightness of the pose averaging problem with increasing measurement noise level, *σ*. At each noise, we conducted 1000 trials of averaging 10 noisy poses. (left) We see that the local solver (randomly initialized) finds the global minimum with decreasing frequency (green) as the measurement noise is increased, while the SDP solver (blue) successfully produces rank-1 solutions (we consider log SVR of at least 5 to be rank 1) to much higher noise levels. For completeness, we also show how frequently the local solver converges to any minimum (red). (right) Boxplots of the log SVR of the SDP solution show that the global solution remains highly rank 1 over a wide range of measurement noise values.

The appendix again provides a baseline local solver for this problem. For the global (SDP) solver we used cvx in Matlab with mosek (ApS, 2019). The solution costs of the global and local solvers agree to high precision if a good initial guess is given to the local solver. Figure 4 provides examples of the local solver becoming trapped in poor local minima while the global solver converges to the correct minima near the groundtruth. Figure 5 provides a quantitative study of the tightness of the SDP solution with increasing measurement noise; we selected the measurement covariance as $W_{m}^{-1}=\sigma^{2}I$ , with *σ* increasing. We again see there is a large range for the noise over which the local solver can become trapped in a local minimum while the global solver remains tight. With *M* = 10 poses to be averaged, the local solver took on average 0.0064*s* while the SDP solver took on average 0.5944*s*.

**Figure 6.**
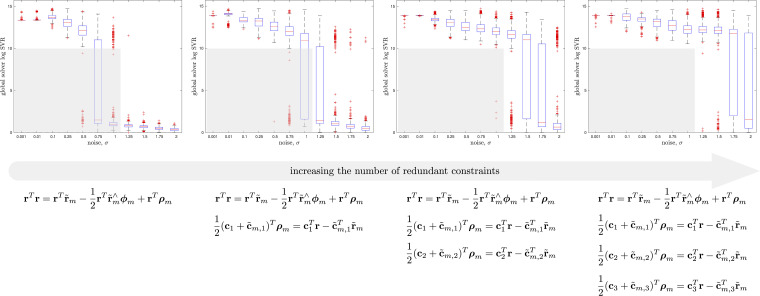
*Pose Averaging Ablation Study:* Here we show the effect on SDP tightness of varying the number of redundant constraints in the pose averaging problem. The rightmost column shows our full set of recommended redundant constraints with the light-grey box indicating the region of measurement noise for which our problem can be deemed tight. The same grey box is shown in the other columns for reference, indicating that including fewer redundant constraints results in a lower level of noise for which we can keep the solution tight.

To justify the need for the redundant constraints, we conducted an ablation study (see Figure 6) wherein we varied the number of redundant constraints. The study shows that with more redundant constraints, we can tolerate a higher level of measurement noise while keeping the SDP tight. We always included the last redundant constraint in (36) as this enforces that the search space for the SDP remains compact^7^ and it is therefore well posed. In the interest of space, we forgo similar ablation studies for the subsequent problems (discrete-time and continuous-time trajectory estimation), which reuse the pose averaging redundant constraints and then build on top of them. The studies are similar in that the more redundant constraints we add, the larger the noise region for which we can a priori predict that we will achieve rank-1 SDP solutions.

## 4. Discrete-time trajectory estimation

**Figure 7.**
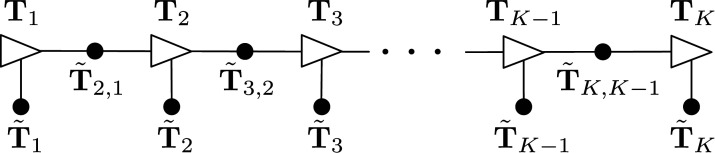
*Discrete-time Trajectory Estimation:* Factor graph representation of the discrete-time estimation problem. Each black dot represents one of the error terms in the cost function of (37).

Our next problem is to consider estimation of a trajectory of *K* poses, **T**_*k*_, where we have noisy measurements of each pose, ${\tilde{T}}_{k}$ , as well as noisy relative measurements, ${\tilde{T}}_{k+1,k}$ , from one pose to the next. The optimization problem that we want to solve is

(37) $$\begin{array}{ccc} \min & \overset{K}{\underset{k=1}{\sum }}{\text{cay}}^{-1}{\left(T_{k}{\tilde{T}}_{k}^{-1}\right)}^{\vee^{T}}W_{k}\;{\text{cay}}^{-1}{\left(T_{k}{\tilde{T}}_{k}^{-1}\right)}^{\vee } & \\ & \;+\;\overset{K-1}{\underset{k=1}{\sum }}{\text{cay}}^{-1}{\left(T_{k+1}T_{k}^{-1}{\tilde{T}}_{k+1,k}^{-1}\right)}^{\vee^{T}} & \\ & \;\;\times \;W_{k+1,k}\;{\text{cay}}^{-1}{\left(T_{k+1}T_{k}^{-1}{\tilde{T}}_{k+1,k}^{-1}\right)}^{\vee } & \\ w.r.t. & T_{k}\;\left(\forall k\right) & \\ s.t. & T_{k}\in SE\left(3\right)\;(\forall k), & \end{array}$$

for some weight matrices, **W**_*k*_ and **W**_*k*+1,*k*_. Figure 7 depicts the estimation problem as a factor graph. Similarly to the pose averaging problem, we introduce new optimization variables for the residual errors:

(38a) $$\begin{array}{ll} \xi_{k} & ={\text{cay}}^{-1}{\left(T_{k}{\tilde{T}}_{k}^{-1}\right)}^{\vee }, \end{array}$$

(38b) $$\begin{array}{ll} \xi_{k+1,k} & ={\text{cay}}^{-1}{\left(T_{k+1}T_{k}^{-1}{\tilde{T}}_{k+1,k}^{-1}\right)}^{\vee }, \end{array}$$

so that the optimization problem can be stated as a QCQP:

(39) $$\begin{array}{ccc} \min & \overset{K}{\underset{k=1}{\sum }}\xi_{k}^{T}W_{k}\xi_{k}+\overset{K-1}{\underset{k=1}{\sum }}\xi_{k+1,k}^{T}W_{k+1,k}\xi_{k+1,k} & \\ w.r.t. & T_{k},\xi_{k},\xi_{k+1,k}\;\left(\forall k\right) & \\ s.t. & C_{k}^{T}C_{k}=I\;\left(\forall k\right) & \\ & \left(I-\frac{1}{2}\xi_{k}^{\wedge }\right)T_{k}=\left(I+\frac{1}{2}\xi_{k}^{\wedge }\right){\tilde{T}}_{k}\;\left(\forall k\right) & \\ & \left(I-\frac{1}{2}\xi_{k+1,k}^{\wedge }\right)T_{k+1} & \\ & \;\;=\left(I+\frac{1}{2}\xi_{k+1,k}^{\wedge }\right){\tilde{T}}_{k+1,k}T_{k}\;(\forall k), & \end{array}$$

where the det(**C**_*k*_) = 1 constraints have been dropped. Decomposing the matrices according to

(40a) $$\begin{array}{l} T_{k}=\left[\begin{array}{cc} C_{k} & r_{k}\\ 0^{T} & 1 \end{array}\right]=\left[\begin{array}{cccc} c_{k,1} & c_{k,2} & c_{k,3} & r_{k}\\ 0 & 0 & 0 & 1 \end{array}\right], \end{array}$$

(40b) $$\begin{array}{l} {\tilde{T}}_{k}=\left[\begin{array}{cc} {\tilde{C}}_{k} & {\tilde{r}}_{k}\\ 0^{T} & 1 \end{array}\right]=\left[\begin{array}{cccc} {\tilde{c}}_{k,1} & {\tilde{c}}_{k,2} & {\tilde{c}}_{k,2} & {\tilde{r}}_{k}\\ 0 & 0 & 0 & 1 \end{array}\right], \end{array}$$

(40c) $$\begin{array}{l} {\tilde{T}}_{k+1,k}=\left[\begin{array}{cc} {\tilde{C}}_{k+1,k} & {\tilde{r}}_{k+1,k}\\ 0^{T} & 1 \end{array}\right] \end{array}$$

(40d) $$\begin{array}{l} \;=\left[\begin{array}{cccc} {\tilde{c}}_{k+1,k,1} & {\tilde{c}}_{k+1,k,2} & {\tilde{c}}_{k+1,k,2} & {\tilde{r}}_{k+1,k}\\ 0 & 0 & 0 & 1 \end{array}\right], \end{array}$$

(40e) $$\begin{array}{l} \xi_{k}=\left[\begin{array}{c} \rho_{k}\\ \phi_{k} \end{array}\right],\;\xi_{k+1,k}=\left[\begin{array}{c} \rho_{k+1,k}\\ \phi_{k+1,k} \end{array}\right], \end{array}$$

the QCQP optimization problem can be rewritten compactly as

(41) $$\begin{array}{ccc} \min & \overset{K}{\underset{k=1}{\sum }}\xi_{k}^{T}W_{k}\xi_{k}+\overset{K-1}{\underset{k=1}{\sum }}\xi_{k+1,k}^{T}W_{k+1,k}\xi_{k+1,k} & \\ w.r.t. & c_{k,i},r_{k},\rho_{k},\phi_{k},\rho_{k+1,k},\phi_{k+1,k}\;\left(\forall i,k\right) & \\ s.t. & c_{k,i}^{T}c_{k,j}=\delta_{ij}\;\left(\forall i,j,k\right) & \\ & \left(I-\frac{1}{2}\phi_{k}^{\wedge }\right)c_{k,i}=\left(I-\frac{1}{2}\phi_{k}^{\wedge }\right){\tilde{c}}_{k,i}\;\left(\forall i,k\right) & \\ & \left(I-\frac{1}{2}\phi_{k}^{\wedge }\right)r_{k}=\left(I+\frac{1}{2}\phi_{k}^{\wedge }\right){\tilde{r}}_{k}+\rho_{k}\;\left(\forall k\right) & \\ & \left(I-\frac{1}{2}\phi_{k+1,k}^{\wedge }\right)c_{k+1,i} & \\ & \;=\left(I-\frac{1}{2}\phi_{k+1,k}^{\wedge }\right){\tilde{C}}_{k+1,k}c_{k,i}\;\left(\forall i,k\right) & \\ & \left(I-\frac{1}{2}\phi_{k+1,k}^{\wedge }\right)r_{k+1} & \\ & \;=\left(I+\frac{1}{2}\phi_{k+1,k}^{\wedge }\right)\left({\tilde{C}}_{k+1,k}r_{k}+{\tilde{r}}_{k+1,k}\right) & \\ & \;\;+\;\rho_{k+1,k}\;\left(\forall k\right). & \end{array}$$

Similarly to the pose averaging problem, if we convert this QCQP to a SDP, it is not always tight even for low-noise levels. We need to include some redundant constraints to improve tightness. For each of the ***ξ***_*k*_ variables, we can create copies of the redundant constraints required in the pose averaging problem. However, this is still not enough; we require some additional constraints involving the ***ξ***_*k*+1,*k*_ variables.

Such additional redundant constraints can be formed by combining the last two of (41); the second last can be written as

(42) $$\begin{array}{l} \frac{1}{2}{\left(c_{k+1,i}+{\tilde{C}}_{k+1,k}c_{k,i}\right)}^{T}\phi_{k+1,k}^{\wedge }\\ =-{\left(c_{k+1,i}-{\tilde{C}}_{k+1,k}c_{k,i}\right)}^{T}, \end{array}$$

while the last can be premultiplied by ${\left(c_{k+1,i}+{\tilde{C}}_{k+1,k}c_{k,i}\right)}^{T}$ and written as

(43) $$\begin{array}{l} {\left(c_{k+1,i}+{\tilde{C}}_{k+1,k}c_{k,i}\right)}^{T}\left(r_{k+1}-{\tilde{C}}_{k+1,k}r_{k}-{\tilde{r}}_{k+1,k}\right)\\ =\underset{-{\left(c_{k+1,i}-{\tilde{C}}_{k+1,k}c_{k,i}\right)}^{T}}{\underbrace{\frac{1}{2}{\left(c_{k+1,i}+{\tilde{C}}_{k+1,k}c_{k,i}\right)}^{T}\phi_{k+1,k}^{\wedge }}}\\ \times \;\left(r_{k+1}+{\tilde{C}}_{k+1,k}r_{k}+{\tilde{r}}_{k+1,k}\right)\\ +{\left(c_{k+1,i}+{\tilde{C}}_{k+1,k}c_{k,i}\right)}^{T}\rho_{k+1,k}. \end{array}$$

After performing the indicated substitution, this becomes

(44) $$\begin{array}{l} \frac{1}{2}{\left(c_{k+1,i}+{\tilde{C}}_{k+1,k}c_{k,i}\right)}^{T}\rho_{k+1,k}\\ =c_{k+1,i}^{T}r_{k+1}-c_{k,i}^{T}\left(r_{k}+{\tilde{C}}_{k+1,k}^{T}{\tilde{r}}_{k+1,k}\right), \end{array}$$

which is once again a quadratic constraint.

Summarizing, the following QCQP offers a reasonably tight SDP relaxation in practice:

(45) $$\begin{array}{ccc} \min & \overset{K}{\underset{k=1}{\sum }}\xi_{k}^{T}W_{k}\xi_{k}+\overset{K-1}{\underset{k=1}{\sum }}\xi_{k+1,k}^{T}W_{k+1,k}\xi_{k+1,k} & \\ w.r.t. & c_{k,i},r_{k},\rho_{k},\phi_{k},\rho_{k+1,k},\phi_{k+1,k}\;\left(\forall i,k\right) & \\ s.t. & c_{k,i}^{T}c_{k,j}=\delta_{ij}\;\left(\forall i,j,k\right) & \\ & \left(I-\frac{1}{2}\phi_{k}^{\wedge }\right)c_{k,i}=\left(I-\frac{1}{2}\phi_{k}^{\wedge }\right){\tilde{c}}_{k,i}\;\left(\forall i,k\right) & \\ & \left(I-\frac{1}{2}\phi_{k}^{\wedge }\right)r_{k}=\left(I+\frac{1}{2}\phi_{k}^{\wedge }\right){\tilde{r}}_{k}+\rho_{k}\;\left(\forall k\right) & \\ & \left(I-\frac{1}{2}\phi_{k+1,k}^{\wedge }\right)c_{k+1,i} & \\ & \;=\left(I-\frac{1}{2}\phi_{k+1,k}^{\wedge }\right){\tilde{C}}_{k+1,k}c_{k,i}\;\left(\forall i,k\right) & \\ & \left(I-\frac{1}{2}\phi_{k+1,k}^{\wedge }\right)r_{k+1} & \\ & \;=\left(I+\frac{1}{2}\phi_{k+1,k}^{\wedge }\right)\left({\tilde{C}}_{k+1,k}r_{k}+{\tilde{r}}_{k+1,k}\right) & \\ & \;\;+\;\rho_{k+1,k}\;\left(\forall k\right) & \\ \left(red.\right) & \frac{1}{2}{\left(c_{k,i}+{\tilde{c}}_{k,i}\right)}^{T}\rho_{k}=c_{k,i}^{T}r_{k}-{\tilde{c}}_{k,i}^{T}{\tilde{r}}_{k}\;\left(\forall i,k\right) & \\ & r_{k}^{T}r_{k}=r_{k}^{T}{\tilde{r}}_{k}-\frac{1}{2}r_{k}^{T}{\tilde{r}}_{k}^{\wedge }\phi_{k}+r_{k}^{T}\rho_{k}\;\left(\forall k\right) & \\ & \frac{1}{2}{\left(c_{k+1,i}+{\tilde{C}}_{k+1,k}c_{k,i}\right)}^{T}\rho_{k+1,k} & \\ & \;=c_{k+1,i}^{T}r_{k+1} & \\ & \;\;-\;c_{k,i}^{T}\left(r_{k}+{\tilde{C}}_{k+1,k}^{T}{\tilde{r}}_{k+1,k}\right)\;\left(\forall i,k\right). & \end{array}$$

We again leave it to the reader to manipulate this into the standard form of (21).

**Figure 8.**
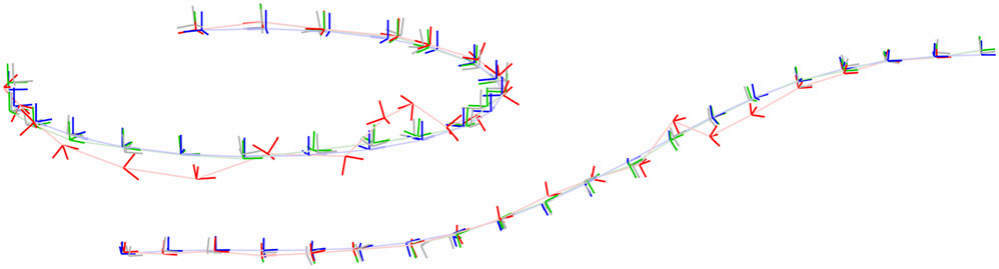
*Discrete-time Trajectory Estimation:* Two examples of discrete-time trajectory estimation where the randomly initialized local solver (red) becomes trapped in a poor local minimum while the global solver (green) finds the correct solution, which is closer to the groundtruth (blue). The noisy pose measurements are also shown (grey). It is interesting to note that the poor local solver solutions are twisted around the groundtruth.

**Figure 9.**
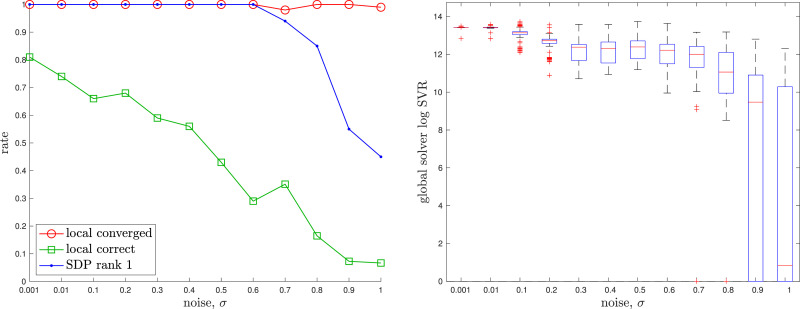
*Discrete-time Trajectory Estimation:* A quantitative evaluation of the tightness of the discrete-time trajectory estimation problem with increasing measurement noise, *σ*. At each noise level, we conducted 100 trials with the geometry of the trajectory as in the left example of Figure 8. (left) We see that the local solver (randomly initialized) finds the global minimum with decreasing frequency (green) as the measurement noise is increased, while the SDP solver (blue) successfully produces rank-1 solutions (we consider log SVR of at least 5 to be rank 1) to much higher noise levels. For completeness, we also show how frequently the local solver converges to any minimum (red). (right) Boxplots of the log SVR of the SDP solution show that the global solution remains highly rank 1 over a wide range of measurement noise values.

The appendix provides a baseline local solver for this problem. For the global (SDP) solver we used cvx in Matlab with mosek (ApS, 2019). The solution costs of the global and local solvers agree to high precision if a good initial guess is given to the local solver. Figure 8 provides examples of the local solver becoming trapped in poor local minima while the global solver converges to the correct minima near the groundtruth. Figure 9 provides a quantitative study of the tightness of the SDP solution with increasing measurement noise; we selected the measurement covariances as $W_{k}^{-1}=W_{k+1,k}^{-1}=\sigma^{2}I$ , with *σ* increasing. We again see there is a large range for the noise over which the local solver can become trapped in a local minimum while the global solver remains tight; in fact, even at very low-noise levels it is quite easy to have the local solver become trapped. With *K* = 20 poses in the trajectory, the local solver took on average 0.1574*s* while the SDP solver took on average 14.32*s*.

## 5. Continuous-time trajectory estimation

**Figure 10.**
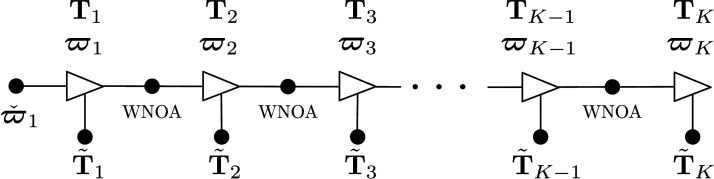
Factor graph representation of the continuous-time estimation problem. Each block dot represents one of the error terms in the cost function of (46).

Finally, we consider so-called continuous-time trajectory estimation. Continuous-time methods come in parametric (Furgale et al., 2012) and nonparametric (Anderson and Barfoot, 2015; Barfoot et al., 2014) varieties; here we will discuss the latter. We consider a continuous-time Gaussian Process (GP) prior over the trajectory known as White Noise on Acceleration (WNOA); this serves to smooth the trajectory and is fused with pose measurements provided at discrete times. We will still ultimately have to discretize the trajectory for the purpose of estimation and so will have *K* states comprising both pose and generalized velocity (a.k.a., twist), $\left\{T_{k},\varpi_{k}\right\}$ . Figure 10 depicts the situation as a factor graph. In practice, we may not actually have pose measurements at every time at which we introduce a state variable.

The optimization problem that we want to solve in this case is

(46) $$\begin{array}{ccc} \min & \sum_{k=1}^{K}{\text{cay}}^{-1}{\left(T_{k}{\tilde{T}}_{k}^{-1}\right)}^{\vee^{T}}W_{k}\;{\text{cay}}^{-1}{\left(T_{k}{\tilde{T}}_{k}^{-1}\right)}^{\vee } & \\ & \;+\;{\left({\overset{ˇ}{\varpi }}_{1}-\varpi_{1}\right)}^{T}Q_{1}^{-1}\left({\overset{ˇ}{\varpi }}_{1}-\varpi_{1}\right) & \\ & \;+\;\sum_{k=1}^{K-1}e_{k+1,k}^{T}Q_{k+1,k}^{-1}\;e_{k+1,k} & \\ w.r.t. & T_{k},\varpi_{k}\;\left(\forall k\right) & \\ s.t. & T_{k}\in SE\left(3\right)\;\left(\forall k\right), & \end{array}$$

for some weight matrices, **W**_*k*_, and

(47a) $$\begin{array}{l} e_{k+1,k}=\left[\begin{array}{c} \left(t_{k+1}-t_{k}\right)\varpi_{k}-{\text{cay}}^{-1}{\left(T_{k+1}T_{k}^{-1}\right)}^{\vee }\\ \varpi_{k}-\varpi_{k+1} \end{array}\right], \end{array}$$

(47b) $$\begin{array}{l} Q_{k+1,k}=\left[\begin{array}{cc} \frac{1}{3}{\left(t_{k+1}-t_{k}\right)}^{3}Q_{c} & \frac{1}{2}{\left(t_{k+1}-t_{k}\right)}^{2}Q_{c}\\ \frac{1}{2}{\left(t_{k+1}-t_{k}\right)}^{2}Q_{c} & \left(t_{k+1}-t_{k}\right)Q_{c} \end{array}\right]. \end{array}$$

The *t*_*k*_ are known timestamps of the states, **Q**_*c*_ is a power-spectral density matrix affecting smoothness of the GP prior, and ${\overset{ˇ}{\varpi }}_{1}$ together with **Q**_1_ represent a Gaussian prior on the initial generalized velocity. The GP prior defined by (47a) is similar in spirit to the one first defined by Anderson and Barfoot (2015), only now adapted to work with the Cayley map. Looking at **e**_*k*+1,*k*_, the first row encourages the change in pose from one time to the next to be similar to the generalized velocity multiplied by the change in time; the second row encourages the generalized velocity to remain constant over time (i.e., no acceleration). The process noise covariance, **Q**_*k*+1,*k*_, comes from integrating the WNOA prior over the same time interval (Barfoot, 2024; Barfoot et al., 2014).

Similarly to the discrete-time trajectory estimation case, we introduce the following substitution variables^8^:

(48a) $$\begin{array}{ll} \xi_{k} & ={\text{cay}}^{-1}{\left(T_{k}{\tilde{T}}_{k}^{-1}\right)}^{\vee }, \end{array}$$

(48b) $$\begin{array}{ll} \xi_{k+1,k} & ={\text{cay}}^{-1}{\left(T_{k+1}T_{k}^{-1}\right)}^{\vee }, \end{array}$$

so that the optimization problem can be stated as a QCQP:

(49) $$\begin{array}{ccc} \min & \sum_{k=1}^{K}\xi_{k}^{T}W_{k}\xi_{k}+{\left({\overset{ˇ}{\varpi }}_{1}-\varpi_{1}\right)}^{T}Q_{1}^{-1}\left({\overset{ˇ}{\varpi }}_{1}-\varpi_{1}\right) & \\ & \;+\;\sum_{k=1}^{K-1}{\left[\begin{array}{c} \left(t_{k+1}-t_{k}\right)\varpi_{k}-\xi_{k+1,k}\\ \varpi_{k}-\varpi_{k+1} \end{array}\right]}^{T} & \\ & \;\;\times Q_{k+1,k}^{-1}\;\left[\begin{array}{c} \left(t_{k+1}-t_{k}\right)\varpi_{k}-\xi_{k+1,k}\\ \varpi_{k}-\varpi_{k+1} \end{array}\right] & \\ w.r.t. & T_{k},\varpi_{k},\xi_{k},\xi_{k+1,k}\;\left(\forall k\right) & \\ s.t. & C_{k}^{T}C_{k}=I\;\left(\forall k\right) & \\ & \left(I-\frac{1}{2}\xi_{k}^{\wedge }\right)T_{k}=\left(I+\frac{1}{2}\xi_{k}^{\wedge }\right){\tilde{T}}_{k}\;\left(\forall k\right) & \\ & \left(I-\frac{1}{2}\xi_{k+1,k}^{\wedge }\right)T_{k+1}=\left(I+\frac{1}{2}\xi_{k+1,k}^{\wedge }\right)T_{k}\;\left(\forall k\right), & \end{array}$$

where the det(**C**_*k*_) = 1 constraints have been dropped. Decomposing the matrices according to

(50a) $$\begin{array}{l} T_{k}=\left[\begin{array}{cc} C_{k} & r_{k}\\ 0^{T} & 1 \end{array}\right]=\left[\begin{array}{cccc} c_{k,1} & c_{k,2} & c_{k,3} & r_{k}\\ 0 & 0 & 0 & 1 \end{array}\right], \end{array}$$

(50b) $$\begin{array}{l} {\tilde{T}}_{k}=\left[\begin{array}{cc} {\tilde{C}}_{k} & {\tilde{r}}_{k}\\ 0^{T} & 1 \end{array}\right]=\left[\begin{array}{cccc} {\tilde{c}}_{k,1} & {\tilde{c}}_{k,2} & {\tilde{c}}_{k,2} & {\tilde{r}}_{k}\\ 0 & 0 & 0 & 1 \end{array}\right], \end{array}$$

(50c) $$\begin{array}{l} \xi_{k}=\left[\begin{array}{c} \rho_{k}\\ \phi_{k} \end{array}\right],\;\xi_{k+1,k}=\left[\begin{array}{c} \rho_{k+1,k}\\ \phi_{k+1,k} \end{array}\right], \end{array}$$

the QCQP optimization problem can be rewritten compactly as

(51) $$\begin{array}{ccc} \min & \sum_{k=1}^{K}\xi_{k}^{T}W_{k}\xi_{k}+{\left({\overset{ˇ}{\varpi }}_{1}-\varpi_{1}\right)}^{T}Q_{1}^{-1}\left({\overset{ˇ}{\varpi }}_{1}-\varpi_{1}\right) & \\ & \;+\;\sum_{k=1}^{K-1}{\left[\begin{array}{c} \left(t_{k+1}-t_{k}\right)\varpi_{k}-\xi_{k+1,k}\\ \varpi_{k}-\varpi_{k+1} \end{array}\right]}^{T} & \\ & \;\;\times Q_{k+1,k}^{-1}\;\left[\begin{array}{c} \left(t_{k+1}-t_{k}\right)\varpi_{k}-\xi_{k+1,k}\\ \varpi_{k}-\varpi_{k+1} \end{array}\right] & \\ w.r.t. & c_{k,i},r_{k},\rho_{k},\phi_{k},\rho_{k+1,k},\phi_{k+1,k},\varpi_{k}\;\left(\forall i,k\right) & \\ s.t. & c_{k,i}^{T}c_{k,j}=\delta_{ij}\;\left(\forall i,j,k\right) & \\ & \left(I-\frac{1}{2}\phi_{k}^{\wedge }\right)c_{k,i}=\left(I-\frac{1}{2}\phi_{k}^{\wedge }\right){\tilde{c}}_{k,i}\;\left(\forall i,k\right) & \\ & \left(I-\frac{1}{2}\phi_{k}^{\wedge }\right)r_{k}=\left(I+\frac{1}{2}\phi_{k}^{\wedge }\right){\tilde{r}}_{k}+\rho_{k}\;\left(\forall k\right) & \\ & \left(I-\frac{1}{2}\phi_{k+1,k}^{\wedge }\right)c_{k+1,i} & \\ & \;=\;\left(I-\frac{1}{2}\phi_{k+1,k}^{\wedge }\right)c_{k,i}\;\left(\forall i,k\right) & \\ & \left(I-\frac{1}{2}\phi_{k+1,k}^{\wedge }\right)r_{k+1} & \\ & \;=\;\left(I+\frac{1}{2}\phi_{k+1,k}^{\wedge }\right)r_{k}+\rho_{k+1,k}\;\left(\forall k\right). & \end{array}$$

Similarly to the problems above, if we convert this QCQP to a SDP, it is not tight even for low-noise levels. We need to include some redundant constraints to improve tightness. For each of the ***ξ***_*k*_ and ***ξ***_*k*+1,*k*_ variables, we can create copies of the redundant constraints required in the discrete-time trajectory estimation problem. However, this is still not always enough to tighten the problem, particularly when we do not have a pose measurement at every state time (see below comment regarding sparser measurement graphs).

We can generate some additional redundant constraints fairly easily. First, we can premultiply the second constraint of (51) by $\phi_{k}^{T}$ so that

(52) $$\begin{array}{l} \phi_{k}^{T}\left(I-\frac{1}{2}\phi_{k}^{\wedge }\right)c_{k,i}=\phi_{k}^{T}\left(I-\frac{1}{2}\phi_{k}^{\wedge }\right){\tilde{c}}_{k,i}\\ \Rightarrow \;\phi_{k}^{T}c_{k,i}=\phi_{k}^{T}{\tilde{c}}_{k,i}, \end{array}$$

where we have used that $\phi_{k}^{T}\phi_{k}^{\wedge }=0^{T}$ . Similarly, premultiplying the third constraint in (51) by $\phi_{k}^{T}$ results in

(53) $$\phi_{k}^{T}r_{k}=\phi_{k}^{T}{\tilde{r}}_{k}+\phi_{k}^{T}\rho_{k}.$$

Premultiplying the fourth and fifth constraints in (51) by $\phi_{k+1,k}^{T}$ results in

(54a) $$\begin{array}{l} \phi_{k+1,k}^{T}c_{k+1,i}=\phi_{k+1,k}^{T}c_{k,i}, \end{array}$$

(54b) $$\begin{array}{l} \phi_{k+1,k}^{T}r_{k+1}=\phi_{k+1,k}^{T}r_{k}+\phi_{k+1,k}^{T}\rho_{k+1,k}. \end{array}$$

Next, we can exploit the fact that columns of a rotation matrix satisfy $c_{\ell }^{\wedge }c_{m}=c_{n}$ where $\ell mn\in \left\{123,231,312\right\}$ . If we premultiply the second constraint of (51) by $c_{k,m}^{T}$ we have

(55) $$\begin{array}{l} c_{k,m}^{T}\left(I-\frac{1}{2}\phi_{k}^{\wedge }\right)c_{k,\ell }=c_{k,m}^{T}\left(I-\frac{1}{2}\phi_{k}^{\wedge }\right){\tilde{c}}_{k,\ell }\\ \Rightarrow \;c_{k,m}^{T}c_{k,\ell }-\frac{1}{2}\phi_{k}^{T}c_{k,n}=c_{k,m}^{T}{\tilde{c}}_{k,\ell }-\frac{1}{2}c_{k,m}^{T}{\tilde{c}}_{k,\ell }^{\wedge }\phi_{k}, \end{array}$$

which is still a quadratic constraint. Finally, if we premultiply the last constraint of (51) by ${\left(r_{k+1}+r_{k}\right)}^{T}$ , this results in

(56) $$r_{k+1}^{T}r_{k+1}=r_{k+1}^{T}\rho_{k+1,k}+r_{k}^{T}\rho_{k+1,k}+r_{k}^{T}r_{k,}$$

which is once again a quadratic constraint.

Summarizing, the following QCQP offers a reasonably tight SDP relaxation in practice:

(57) $$\begin{array}{ccc} \min & \sum_{k=1}^{K}\xi_{k}^{T}W_{k}\xi_{k}+{\left({\overset{ˇ}{\varpi }}_{1}-\varpi_{1}\right)}^{T}Q_{1}^{-1}\left({\overset{ˇ}{\varpi }}_{1}-\varpi_{1}\right) & \\ & \;+\;\sum_{k=1}^{K-1}{\left[\begin{array}{c} \left(t_{k+1}-t_{k}\right)\varpi_{k}-\xi_{k+1,k}\\ \varpi_{k}-\varpi_{k+1} \end{array}\right]}^{T} & \\ & \;\;\times Q_{k+1,k}^{-1}\;\left[\begin{array}{c} \left(t_{k+1}-t_{k}\right)\varpi_{k}-\xi_{k+1,k}\\ \varpi_{k}-\varpi_{k+1} \end{array}\right] & \\ w.r.t. & c_{k,i},r_{k},\rho_{k},\phi_{k},\rho_{k+1,k},\phi_{k+1,k},\varpi_{k}\;\left(\forall i,k\right) & \\ s.t. & c_{k,i}^{T}c_{k,j}=\delta_{ij}\;\left(\forall i,j,k\right) & \\ & \left(I-\frac{1}{2}\phi_{k}^{\wedge }\right)c_{k,i}=\left(I-\frac{1}{2}\phi_{k}^{\wedge }\right){\tilde{c}}_{k,i}\;\left(\forall i,k\right) & \\ & \left(I-\frac{1}{2}\phi_{k}^{\wedge }\right)r_{k}=\left(I+\frac{1}{2}\phi_{k}^{\wedge }\right){\tilde{r}}_{k}+\rho_{k}\;\left(\forall k\right) & \\ & \left(I-\frac{1}{2}\phi_{k+1,k}^{\wedge }\right)c_{k+1,i} & \\ & \;=\;\left(I-\frac{1}{2}\phi_{k+1,k}^{\wedge }\right)c_{k,i}\;\left(\forall i,k\right) & \\ & \left(I-\frac{1}{2}\phi_{k+1,k}^{\wedge }\right)r_{k+1} & \\ & \;=\;\left(I+\frac{1}{2}\phi_{k+1,k}^{\wedge }\right)r_{k}+\rho_{k+1,k}\;\left(\forall k\right) & \\ \left(red.\right) & \frac{1}{2}{\left(c_{k,i}+{\tilde{c}}_{k,i}\right)}^{T}\rho_{k}=c_{k,i}^{T}r_{k}-{\tilde{c}}_{k,i}^{T}{\tilde{r}}_{k}\;\left(\forall i,k\right) & \\ & r_{k}^{T}r_{k}=r_{k}^{T}{\tilde{r}}_{k}-\frac{1}{2}r_{k}^{T}{\tilde{r}}_{k}^{\wedge }\phi_{k}+r_{k}^{T}\rho_{k}\;\left(\forall k\right) & \\ & \phi_{k}^{T}c_{k,i}=\phi_{k}^{T}{\tilde{c}}_{k,i}\;\left(\forall i,k\right) & \\ & \phi_{k}^{T}r_{k}=\phi_{k}^{T}{\tilde{r}}_{k}+\phi_{k}^{T}\rho_{k}\;\left(\forall k\right) & \\ & c_{k,m}^{T}c_{k,\ell }-\frac{1}{2}\phi_{k}^{T}c_{k,n} & \\ & \;=c_{k,m}^{T}{\tilde{c}}_{k,\ell }-\frac{1}{2}c_{k,m}^{T}{\tilde{c}}_{k,\ell }^{\wedge }\phi_{k} & \\ & \;\;\;\left(\forall \ell mn\in \left\{123,231,312\right\},k\right) & \\ & \frac{1}{2}{\left(c_{k+1,i}+c_{k,i}\right)}^{T}\rho_{k+1,k} & \\ & \;=\;c_{k+1,i}^{T}r_{k+1}-c_{k,i}^{T}r_{k}\;\left(\forall i,k\right) & \\ & r_{k+1}^{T}r_{k+1} & \\ & \;=\;r_{k+1}^{T}\rho_{k+1,k}+r_{k}^{T}\rho_{k+1,k}+r_{k}^{T}r_{k}\;\left(\forall k\right) & \\ & \phi_{k+1,k}^{T}c_{k+1,i}=\phi_{k+1,k}^{T}c_{k,i}\;\left(\forall i,k\right) & \\ & \phi_{k+1,k}^{T}r_{k+1}=\phi_{k+1,k}^{T}r_{k}+\phi_{k+1,k}^{T}\rho_{k+1,k}\;\left(\forall k\right). & \end{array}$$

We again leave it to the reader to manipulate this into the standard form of (21). We can also notice that the ***ϖ***_*k*_ variables are not involved in any of the constraints and thus remain unconstrained variables. Similar to Rosen et al. (2019); Holmes and Barfoot (2023), at implementation we use the Schur complement to marginalize these variables out of the problem, thereby keeping the size of the SDP as small as possible; we can easily compute them after the main solve.

**Figure 11.**
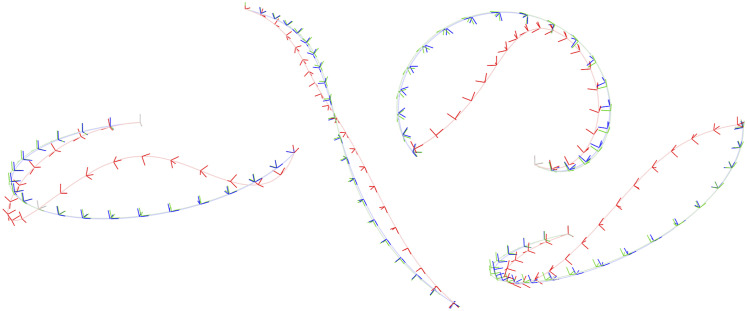
*Continuous-time Trajectory Estimation:* Four examples of continuous-time trajectory estimation where the randomly initialized local solver (red) becomes trapped in a poor local minimum while the global solver (green) finds the correct solution, which is closer to the groundtruth (blue). The noisy pose measurements (occurring only at the start, middle, and end of each trajectory) are also shown (grey). The local minimum in the leftmost example is very similar to one reported by Lilge et al. (2022).

**Figure 12.**
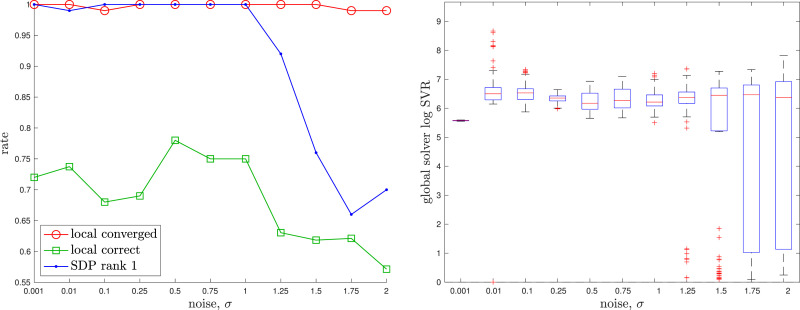
*Continuous-time Trajectory Estimation:* A quantitative evaluation of the tightness of the continuous-time trajectory estimation problem with increasing measurement noise, *σ*. At each noise level, we conducted 100 trials with the geometry of the trajectory as in the left example of Figure 11. (left) We see that the local solver (randomly initialized) finds the global minimum with decreasing frequency (green) as the measurement noise is increased, while the SDP solver (blue) successfully produces rank-1 solutions (we consider log SVR of at least 5 to be rank 1) to much higher noise levels. For completeness, we also show how frequently the local solver converges to any minimum (red). (right) Boxplots of the log SVR of the SDP solution show that the global solution remains reasonably rank 1 over a wide range of measurement noise values.

The appendix provides a baseline local solver for this problem. For the global (SDP) solver we used cvx in Matlab with mosek (ApS, 2019). The solution costs of the global and local solvers agree to high precision if a good initial guess is given to the local solver. Figure 11 provides examples of the local solver becoming trapped in poor local minima while the global solver converges to the correct minimum near the groundtruth. Figure 12 provides a quantitative study of the tightness of the SDP solution with increasing measurement noise; we selected the measurement covariances as $W_{k}^{-1}=\sigma^{2}I$ , with *σ* increasing. We again see there is a large range for the noise over which the local solver can become trapped in a local minimum while the global solver remains tight; in fact, even at very low-noise levels it is quite easy to have the local solver become trapped. With *K* = 21 poses in the trajectory, the local solver took on average 0.1928*s* while the SDP solver took on average 13.42*s*.

There are also a few noteworthy differences in the continuous-time experiment as compared to the discrete-time case. First, the log SVR values are quite a bit lower in Figure 12 as compared to Figure 9. This seems to be mainly due to the fact that we are now using a sparser set of measurements. Our continuous-time experiments had pose measurements only at the start, middle, and end, whereas the discrete-time case had pose measurements at every timestep. It is known that a sparser measurement graph can impact SDP tightness (Holmes and Barfoot, 2023). Second, the low-noise test cases experienced some numerical issues with getting the SDP solver to reliably converge. It seems this is related to matrix conditioning resulting from the fact that we are marginalizing out the ***ϖ***_*k*_ variables before solving the SDP. We found it was necessary to adjust the scaling of the **Q** matrix in (21) to get reliable solutions. Despite our best efforts, we see that there was one out of 100 test cases at the *σ* = 0.01 noise level where the SDP failed to solve. On the log SVR plot this shows as a red plus sign at 0 and results in the success rate of the SDP solver being 0.99 instead of 1. Still, we *know* that the solver failed and therefore not to trust the answer. Overall, we still have log SVR values almost always above 5 up to about *σ* = 1, which makes the solution practical; it is easy for the local solver to become stuck in local minima in this noise range.

## 6. Conclusion and future work

We have presented several new convex relaxations for pose and rotation estimation problems based on the Cayley map. Our results indicate that for small problem sizes, we can successfully achieve global optimality with realistic amounts of noise and even with measurement sparsity in the case of continuous-time trajectory estimation. In each of the experiments, we indicated that covariance of the error associated with each pose measurement cost term is *σ*^2^**I**. In other words, *σ* is the standard deviation of the measurement noise. In the case of rotational degrees of freedom, *σ* = 0.5 is already shown in Figure 1 to represent quite a lot of rotational uncertainty, indeed more than typically occurs in practice. Since the standard deviation is in radians, this implies that uncertainty spreads out over a large part of a full circle with *σ* = 0.5. The fact that our convex relaxations empirically remain tight (ensuring global optimality) beyond the *σ* = 0.5 level (often beyond *σ* = 1) means our technique works over most practical situations. For translational degrees of freedom, *σ* will have units of distance. The trajectories in the examples have poses that are spaced one distance unit apart so when the noise on the measurement of one of these poses is *σ* = 0.5 distance units, that is again quite a lot of noise in comparison to the spacing of the poses. The implication is again that our convex relaxations remain tight (ensuring global optimality) for most practical situations.

While our results are promising, we are still relying on off-the-shelf solvers once our problem has been converted to an SDP, which means that we will not be able to scale up to extremely large state sizes. To scale up, there are a few possibilities that we could explore. First, perhaps we might be satisfied with merely certifying our local solver solutions. Other works have focussed on this. The challenge is that in most of the problems of this paper, we require redundant constraints to tighten our SDPs. This means that we do not meet the technical condition of Linearly Independent Constraint Qualification (LICQ) (Boyd and Vandenberghe, 2004). It turns out that this makes it more challenging to calculate an optimality certificate. Yang et al. (2021) is a practical example where a certificate has been constructed for this type of situation, but there are still scaling issues. Another possibility is to solve our problems globally using the approach of Burer and Monteiro (2005) (studied more recently by Boumal et al. (2016)). This was exploited with very impressive results by Rosen et al. (2019); Dellaert et al. (2020); however, these problems enjoyed LICQ. To our knowledge, this approach has not been applied to problems in robotics with redundant constraints. Or, perhaps generic SDP solvers can be made to better exploit problem-specific structure (e.g., chordal sparsity). We plan to explore these and other ways of scaling global optimality for a larger range of state estimation problems.

## Figures and Tables

**Figure 13.**
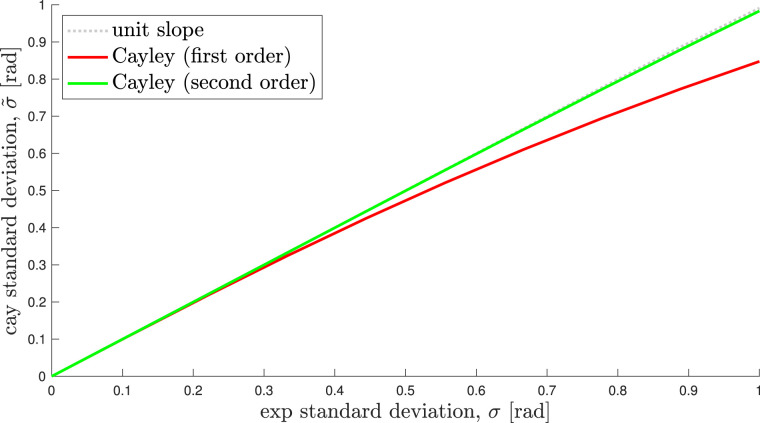
Comparison of the resulting standard deviation of rotational uncertainty between the exp map (horizontal axis) and cay map (vertical) axis. Red (first order) shows the naive approach of letting **Σ**_2_ = **Σ**_1_ and green (second order) shows the improvement offered by (64).

## Notes

1. Some authors instead use the term ‘rotation averaging’, whereas we use rotation averaging to mean the process of fusing several noisy measurements of a single rotation into an estimate.

2. While rank(**X**) = 1 provides an a posteriori guarantee that our solution is globally optimal, we cannot a priori guarantee that we will get a rank-one solution for a given set of measurements. Measurement noise, for example, can have a big impact on tightness and so we will later use empirical studies to a priori gauge how likely we are to find rank-one solutions at different measurement noise levels.

3. The interested reader is directed to Henrion et al., (2021); Lasserre, (2010) for a more in depth treatment on the *moment/sum-of-squares hierarchy*.

4. On each box, the central mark is the median, the edges of the box are the 25th and 75th percentiles, the whiskers extend to the most extreme datapoints the algorithm considers to be not outliers, and the outliers are plotted individually (as red + symbols).

5 This is also a relaxation of the problem, but one that has been shown to often be tight in practice (Rosen et al. 2019).

6. For this and all subsequent experiments, the local solver was verified to be converged in each case to a step size change of less than 10^−6^ while the global solver reported its own solution quality and used the cvx_precision high setting (see the cvx documentation).

7. Compactness in this context is related to the Archimedean property of the feasible set, which is a required condition when tightening a relaxation using Lasserre’s hierarchy (Lasserre, 2010).

8. Importantly, we were forced to deviate from our program of substitution variables being the residual errors of the cost function in our choice of ***ξ***_*k*+1,*k*_; this could be problematic for very-high-velocity trajectories as ***ξ***_*k*+1,*k*_ could become large and approach the singularity of the Cayley map. We justify our choice as providing the necessary pathway to a QCQP, but this could be revisited in future work.

9. The notation was chosen to match the continuous-time estimation chapter of Barfoot (2024).

## Appendix

### A. Comparing exponential and Cayley distributions

We use this section to analyze how similar our proposed Gaussian-like distribution via the Cayley map is to one via the exponential map.

Consider two distributions for rotations, **C**_1_ and **C**_2_, defined according to

(58a)
$$\begin{array}{ll} C_{1} & =\exp\left(\phi_{1}^{\wedge }\right)\bar{C},\;\phi_{1}∼N\left(0,\Sigma_{1}\right), \end{array}$$

(58b)
$$\begin{array}{ll} C_{2} & =\text{cay}\left(\phi_{2}^{\wedge }\right)\bar{C},\;\phi_{2}∼N\left(0,\Sigma_{2}\right). \end{array}$$

To ensure that **C**_1_ = **C**_2_ (in every instance), Bauchau and Trainelli (2003) show that we require

(59a)
$$\begin{array}{ll} \phi_{1} & =\phi_{1}\;a\;=\;\varphi \;a, \end{array}$$

(59b)
$$\begin{array}{ll} \phi_{2} & =\phi_{2}\;a\;=2\tan\left(\frac{\varphi }{2}\right)\;a, \end{array}$$

in terms of a common axis of rotation, **a**, and angle of rotation, *φ*. Notationally, we define *ϕ*_1_ = ‖ ***ϕ***_1_‖ = *φ* and $\phi_{2}=\|\phi_{2}\|=2\tan\left(\frac{\varphi }{2}\right)$ . Rearranging, we therefore require

(60)
$$\phi_{2}=\frac{\tan\left(\frac{\phi_{1}}{2}\right)}{\frac{\phi_{1}}{2}}\phi_{1}$$

to relate ***ϕ***_1_ and ***ϕ***_2_ directly.

We can use (60) to relate the distribution for ***ϕ***_2_ to that of ***ϕ***_1_, which will in turn make the distribution for **C**_2_ approximately match that of **C**_1_. Specifically, we will try to match the moments of the left and right sides of (60). Moment matching is known to minimize a version of Kullback-Liebler divergence between two distributions (Bishop, 2006).

Expanding the tan function in (60) using a Taylor series we can write

(61)
$$\phi_{2}=\left(1+\frac{1}{12}\phi_{1}^{T}\phi_{1}+\frac{1}{120}{\left(\phi_{1}^{T}\phi_{1}\right)}^{2}+\cdots \;\right)\phi_{1}.$$

We have only odd powers of ***ϕ***_1_ appearing on the right side of this expression so we immediately see that we want

(62)
$$E\left[\phi_{2}\right]=0.$$

This does not involve any approximation. We only need to use that ***ϕ***_1_ follows a Gaussian distribution, which has all its odd moments zero.

For the covariance matrix, $\Sigma_{2}=E\left[\phi_{2}\phi_{2}^{T}\right]$ , we can insert (61) and truncate after a number of terms to match the required covariance approximately. For example, keeping terms out to quartic in ***ϕ***_1_ we have

(63)
$$\begin{array}{l} \Sigma_{2}\approx E\left[\phi_{1}\phi_{1}^{T}\right]+\frac{1}{6}E\left[\phi_{1}\phi_{1}^{T}\phi_{1}\phi_{1}^{T}\right]\\ =\Sigma_{1}+\frac{1}{6}\left(\text{tr}\left(\Sigma_{1}\right)I+2\Sigma_{1}\right)\Sigma_{1}, \end{array}$$

where we have employed Isserlis’ theorem (Barfoot, 2024, §2.2.17) to compute the expectation on the right. Thus defining

(64)
$$\phi_{2}∼N(0,\underset{\Sigma_{2}}{\underbrace{\Sigma_{1}+\frac{1}{6}\left(\text{tr}\left(\Sigma_{1}\right)I+2\Sigma_{1}\right)\Sigma_{1}}})$$

will result in **C**_1_ and **C**_2_ approximately having the same distribution. We will refer to the approximation **Σ**_2_ ≈ **Σ**_1_ as ‘first order’ (in **Σ**_1_) and the better version in (64) as ‘second order’ (in **Σ**_1_). More terms in the approximation could be computed depending on the desired level of accuracy.

To visualize our approach, we can provide a one-dimensional example. Consider $\Sigma_{1}=\mathrm{diag}\left(\sigma_{1}^{2},0,0\right)$ , so the axis of rotation, $a={\left[\begin{array}{ccc} 1 & 0 & 0 \end{array}\right]}^{T}$ , is held fixed. Then the distributions for *ϕ*_1_ and *ϕ*_2_ will be

(65)
$$p_{1}\left(\phi_{1}\right)=N\left(0,\sigma_{1}^{2}\right),\;p_{2}\left(\phi_{2}\right)=N(0,\sigma_{2}^{2}),$$

where either $\sigma_{2}^{2}=\sigma_{1}^{2}$ (first order) or $\sigma_{2}^{2}=\sigma_{1}^{2}+\frac{1}{2}\sigma_{1}^{4}$ (second order using (64)). If we map *ϕ*_1_ and *ϕ*_2_ through exp and cay, respectively, we can produce densities on the actual rotational angle, *φ*:

(66a)
$$\begin{array}{l} exp\;:\;p_{1}\left(\varphi \right)=\frac{1}{\sqrt{2\pi \sigma_{1}^{2}}}\exp\left(-\frac{1}{2}\frac{\varphi^{2}}{\sigma_{2}^{1}}\right), \end{array}$$

(66b)
$$\begin{array}{l} \text{cay}\;:\;p_{2}\left(\varphi \right)=\frac{1}{\sqrt{2\pi \sigma_{2}^{2}}}\exp\left(-\frac{1}{2}\frac{{\left(2\tan\left(\frac{\varphi }{2}\right)\right)}^{2}}{\sigma_{2}^{2}}\right)\sec^{2}\left(\frac{\varphi }{2}\right).\; \end{array}$$

We arrive at these by inserting the substitutions from (59) into the distributions in (65). The exp distribution is straightforward since *ϕ*_1_ = *φ*, which results in a Gaussian. The cay distribution is slightly less obvious so we show the steps. We first note that

(67)
$$\phi_{2}=2\tan\left(\frac{\varphi }{2}\right)\;\Rightarrow \;d\phi_{2}=\sec^{2}\left(\frac{\varphi }{2}\right)\;d\varphi .$$

Then, to continue to satisfy the axiom of total probability we require

(68)
$$\begin{array}{l} 1=\int \underset{p_{2}\left(\phi_{2}\right)}{\underbrace{\frac{1}{\sqrt{2\pi \sigma_{2}^{2}}}\exp\left(-\frac{1}{2}\frac{\phi_{2}^{2}}{\sigma_{2}^{2}}\right)}}\;d\phi_{2}\\ =\int \underset{p_{2}\left(\varphi \right)}{\underbrace{\frac{1}{\sqrt{2\pi \sigma_{2}^{2}}}\exp\left(-\frac{1}{2}\frac{{\left(2\tan\left(\frac{\varphi }{2}\right)\right)}^{2}}{\sigma_{2}^{2}}\right)\sec^{2}\left(\frac{\varphi }{2}\right)}}\;d\varphi . \end{array}$$

We see that *p*_2_(*φ*) is no longer Gaussian in this case. However, the idea is that the variances of *p*_1_(*φ*) and *p*_2_(*φ*) should be close when we compute *σ*_2_ using (64). Figure 1 provides examples when *σ*_1_ = 0.2 [rad] and *σ*_1_ = 0.5 [rad]; despite the latter being a fairly large rotational uncertainty, we see the distributions match quite well; their variances are almost identical.

Figure 13 shows the standard deviation, $\tilde{\sigma }$ , of *φ* using the Cayley map distribution (numerically computed from *p*_2_(*φ*)) as *σ* = *σ*_1_ is increased. We see the standard deviations, *σ* and $\tilde{\sigma }$ , match quite well up to a large amount of rotational uncertainty. These results suggest that replacing instances of the exponential map with the Cayley map may have little effect; depending on the amount of rotational uncertainty, we may choose to ‘correct’ the covariance using (64) or we could even include more terms to improve the approximation.

### B. Local solvers

As the local solvers are not the main subject of the paper, we use this appendix to explain how these were implemented for each of the estimation problems. We begin by outlining the required Lie-group Jacobians.

#### B.1. Jacobians for rotations and poses

In order to devise local optimization algorithms that work with Lie groups, we require the (inverse) Jacobians for *SO*(3) and *SE*(3). For example, for rotations parameterized through the exponential map, **C**(***ϕ***) = exp(***ϕ***^∧^), the inverse left Jacobian expression is well known to be (Barfoot, 2024)

(69)
$$J_{e}{\left(\phi \right)}^{-1}=\frac{\phi }{2}\cot\frac{\phi }{2}I-\frac{\phi }{2}a^{\wedge }+\left(1-\frac{\phi }{2}\cot\frac{\phi }{2}\right)aa^{T}.$$

The rotational kinematics can thus be written in one of two ways:

(70)
$$\dot{C}\left(\phi \right)=\omega^{\wedge }C\left(\phi \right)\;\Leftrightarrow \;\dot{\phi }=J_{e}{\left(\phi \right)}^{-1}\omega ,$$

where ***ω*** is the angular velocity. Naturally, the expression on the right is subject to singularities. Considering an infinitesimal increment of time, the same Jacobian thus allows us to approximate the compounding of two exponentiated vectors $\phi_{1},\phi_{2}\in R^{3}$ as

(71)
$$\exp\left(\phi_{1}^{\wedge }\right)\;\exp\left(\phi_{2}^{\wedge }\right)\approx \exp\left({\left(J_{e}{\left(\phi_{2}\right)}^{-1}\phi_{1}+\phi_{2}\right)}^{\wedge }\right),$$

where ***ϕ***_1_ is assumed to be ‘small’. A similar situation exists for SE(3) poses parametrized by the exponential map, **T**(***ξ***) = exp(***ξ***^∧^). The kinematics can be written in one of two ways:

(72)
$$\dot{T}\left(\xi \right)=\varpi^{\wedge }T\left(\xi \right)\;\Leftrightarrow \;\dot{\xi }=J_{e}{\left(\xi \right)}^{-1}\varpi ,$$

where ***ϖ*** is the generalized velocity or ‘twist’ and $J_{e}{\left(\xi \right)}^{-1}$ is the inverse Jacobian (expression can be found in Barfoot (2024)). Again, this allows us to approximate the compounding of two exponentiated vectors $\xi_{1},\xi_{2}\in R^{6}$ as

(73)
$$\exp\left(\xi_{1}^{\wedge }\right)\;\exp\left(\xi_{2}^{\wedge }\right)\approx \exp\left({\left(J_{e}{\left(\xi_{2}\right)}^{-1}\xi_{1}+\xi_{2}\right)}^{\wedge }\right),$$

where ***ξ***_1_ is assumed to be ‘small’.

To work with the Cayley map (instead of the exponential map), we will require similar Jacobian expressions, which are summarized neatly by Müller (2021). In our notation, for rotations parameterized by the Cayley map, **C**(***ϕ***) = cay(***ϕ***^∧^), the inverse left Jacobian expression is

(74)
$$J_{c}{\left(\phi \right)}^{-1}=I-\frac{1}{2}\phi^{\wedge }+\frac{1}{4}\phi \phi^{T},$$

which is appealing in that it does not involve any trigonometric functions compared to (69). In passing, the left Jacobian itself is the tidy expression,

(75)
$$J_{c}\left(\phi \right)=\frac{1}{1+\frac{1}{4}\phi^{T}\phi }\left(I+\frac{1}{2}\phi^{\wedge }\right).$$

The rotational kinematics can be written as

(76)
$$\dot{C}\left(\phi \right)=\omega^{\wedge }C\left(\phi \right)\;\Leftrightarrow \;\dot{\phi }=J_{c}{\left(\phi \right)}^{-1}\omega ,$$

and the compounding approximation for vectors $\phi_{1},\phi_{2}\in R^{3}$ is

(77)
$$\text{cay}\left(\phi_{1}^{\wedge }\right)\;\text{cay}\left(\phi_{2}^{\wedge }\right)\approx \text{cay}\left({\left(J_{c}{\left(\phi_{2}\right)}^{-1}\phi_{1}+\phi_{2}\right)}^{\wedge }\right),$$

where ***ϕ***_1_ is assumed to be ‘small’. Finally, for poses parameterized by the Cayley map, **T**(***ξ***) = cay(***ξ***^∧^), the kinematics are

(78)
$$\dot{T}\left(\xi \right)=\varpi^{\wedge }T\left(\xi \right)\;\Leftrightarrow \;\dot{\xi }=J_{c}{\left(\xi \right)}^{-1}\varpi ,$$

where ***ϖ*** is the generalized velocity or ‘twist’ and $J_{c}{\left(\xi \right)}^{-1}$ is the inverse Jacobian:

(79a)
$$\begin{array}{l} J_{c}{\left(\xi \right)}^{-1}=I-\frac{1}{2}\xi^{⋏}+\frac{1}{4}\Lambda \left(\xi \xi^{T}\right), \end{array}$$

(79b)
$$\begin{array}{l} \Lambda \left(\xi \xi^{T}\right)=\left[\begin{array}{cc} 0 & \phi^{\wedge }\rho^{\wedge }\\ 0 & \phi \phi^{T} \end{array}\right]. \end{array}$$

Again, we have a nice expression devoid of any trigonometric functions. The compounding approximation for two vectors $\xi_{1},\xi_{2}\in R^{6}$ is then

(80)
$$\text{cay}\left(\xi_{1}^{\wedge }\right)\;\text{cay}\left(\xi_{2}^{\wedge }\right)\approx \text{cay}\left({\left(J_{c}{\left(\xi_{2}\right)}^{-1}\xi_{1}+\xi_{2}\right)}^{\wedge }\right),$$

where ***ξ***_1_ is assumed to be ‘small’.

#### B.2. Rotation averaging

To devise a local solver for (22), we begin with an initial guess, **C**_op_, and perturb it according to

(81)
$$C=\text{cay}\left(\psi^{\wedge }\right)C_{\text{op},}$$

where $\psi \in R^{3}$ is a small unknown perturbation. This ensures that **C** ∈ *SO*(3) during the optimization process. The error in the cost function is then

(82)
$$\begin{array}{l} \phi_{m}={\text{cay}}^{-1}{\left(C{\tilde{C}}_{m}^{T}\right)}^{\vee }={\text{cay}}^{-1}{\left(\text{cay}\left(\psi^{\wedge }\right)C_{\text{op}}{\tilde{C}}_{m}^{T}\right)}^{\vee }\\ \approx J_{c}{\left(\phi_{\text{op},m}\right)}^{-1}\psi +\phi_{\text{op},m}, \end{array}$$

where we have used (77) to arrive at the approximation and defined $\phi_{\text{op},m}={\text{cay}}^{-1}{\left(C_{\text{op}}{\tilde{C}}_{m}^{T}\right)}^{\vee }$ . A quadratic approximation to the cost function in (22) (in terms of the perturbation) is thus

(83)
$$\begin{array}{l} \overset{M}{\underset{m=1}{\sum }}{\left(J_{c}{\left(\phi_{\text{op},m}\right)}^{-1}\psi +\phi_{\text{op},m}\right)}^{T}\\ \times \;W_{m}\left(J_{c}{\left(\phi_{\text{op},m}\right)}^{-1}\psi +\phi_{\text{op},m}\right). \end{array}$$

To minimize this with respect to ***ψ***, we take the derivative and set to zero for an optimum, resulting in the linear system of equations

(84)
$$\begin{array}{l} \left(\overset{M}{\underset{m=1}{\sum }}J_{c}{\left(\phi_{\text{op},m}\right)}^{-T}W_{m}J_{c}{\left(\phi_{\text{op},m}\right)}^{-1}\right)\psi^{⋆}\\ =\overset{M}{\underset{m=1}{\sum }}J_{c}{\left(\phi_{\text{op},m}\right)}^{-T}W_{m}\phi_{\text{op},m}, \end{array}$$

which we solve for the optimal perturbation, ***ψ***^⋆^. We then apply this optimal perturbation to the initial guess according to (81), and iterate the process to convergence. This is effectively Gauss-Newton adapted to work for *SO*(3) and will converge locally in practice.

##### B.3. Pose averaging

A very similar local solver can be devised for (28). We begin with an initial guess, **T**_op_, and perturb it according to

(85)
$$T=\text{cay}\left(\epsilon^{\wedge }\right)T_{\text{op}},$$

where $\epsilon \in R^{6}$ is a small unknown perturbation. This ensures that **T** ∈ *SE*(3) during the optimization process. A quadratic approximation to the cost function in (28) (in terms of the perturbation) is thus

(86)
$$\begin{array}{l} \overset{M}{\underset{m=1}{\sum }}{\left(J_{c}{\left(\xi_{\text{op},m}\right)}^{-1}\epsilon +\xi_{\text{op},m}\right)}^{T}\\ \times \;W_{m}\left(J_{c}{\left(\xi_{\text{op},m}\right)}^{-1}\epsilon +\xi_{\text{op},m}\right), \end{array}$$

where $\xi_{\text{op},m}={\text{cay}}^{-1}{\left(T_{\text{op}}{\tilde{T}}_{m}^{-1}\right)}^{\vee }$ . To minimize this with respect to ***ϵ***, we take the derivative and set to zero for an optimum, resulting in the linear system of equations

(87)
$$\begin{array}{l} \left(\overset{M}{\underset{m=1}{\sum }}J_{c}{\left(\xi_{\text{op},m}\right)}^{-T}W_{m}J_{c}{\left(\xi_{\text{op},m}\right)}^{-1}\right)\epsilon^{⋆}\\ =\overset{M}{\underset{m=1}{\sum }}J_{c}{\left(\xi_{\text{op},m}\right)}^{-T}W_{m}\xi_{\text{op},m}, \end{array}$$

which we solve for the optimal perturbation, ***ϵ***^⋆^. We then apply this optimal perturbation to the initial guess according to (85), and iterate the process to convergence. This is effectively Gauss-Newton adapted to work for *SE*(3) and will converge locally in practice.

##### B.4. Discrete-time trajectory estimation

To devise a local solver for (37), we begin with initial guesses, **T**_op,k_, and perturb them according to

(88)
$$T_{k}=\text{cay}\left(\epsilon_{k}^{\wedge }\right)T_{\text{op,k}},$$

where $\epsilon_{k}\in R^{6}$ are small unknown perturbations. This ensures that **T**_*k*_ ∈ *SE*(3) during the optimization process. A quadratic approximation to the cost function in (37) (in terms of the perturbations) is thus

(89)
$$\begin{array}{l} \overset{K}{\underset{k=1}{\sum }}{\left(J_{c}{\left(\xi_{\text{op},k}\right)}^{-1}\epsilon_{k}+\xi_{\text{op},k}\right)}^{T}W_{k}\left(J_{c}{\left(\xi_{\text{op},k}\right)}^{-1}\epsilon_{k}+\xi_{\text{op},k}\right)\\ +\;\overset{K-1}{\underset{k=1}{\sum }}{\left(J_{c}{\left(\xi_{\text{op},k+1,k}\right)}^{-1}\left(\epsilon_{k+1}-T_{\text{op},k+1,k}\epsilon_{k}\right)+\xi_{\text{op},k+1,k}\right)}^{T}\\ \times \;W_{k+1,k}\left(J_{c}{\left(\xi_{\text{op},k+1,k}\right)}^{-1}\left(\epsilon_{k+1}-T_{\text{op},k+1,k}\epsilon_{k}\right)+\xi_{\text{op},k+1,k}\right), \end{array}$$

where $\xi_{\text{op},k}={\text{cay}}^{-1}{\left(T_{\text{op},k}{\tilde{T}}_{k}^{-1}\right)}^{\vee }$ , $\xi_{\text{op},k+1,k}={\text{cay}}^{-1}{\left(T_{\text{op},k+1}T_{\text{op},k}^{-1}{\tilde{T}}_{k+1,k}^{-1}\right)}^{\vee }$ , and $T_{\text{op},k+1,k}=\text{Ad}\left(T_{\text{op},k+1}T_{\text{op},k}^{-1}\right)$ . We can tidy this up by defining

(90a)
$$\begin{array}{l} H=\left[\begin{array}{cccc} -J_{c}{\left(\xi_{\text{op},1}\right)}^{-1} & 0 & \cdots & 0\\ J_{c}{\left(\xi_{\text{op},2,1}\right)}^{-1}T_{\text{op},2,1} & -J_{c}{\left(\xi_{\text{op},2,1}\right)}^{-1} & \cdots & 0\\ 0 & -J_{c}{\left(\xi_{\text{op},2}\right)}^{-1} & \cdots & 0\\ 0 & J_{c}{\left(\xi_{\text{op},3,2}\right)}^{-1}T_{\text{op},3,2} & \cdots & 0\\ \vdots & \vdots & \ddots & \vdots \\ 0 & 0 & \cdots & -J_{c}{\left(\xi_{\text{op},K}\right)}^{-1} \end{array}\right], \end{array}$$

(90b)
$$\begin{array}{l} \xi_{\text{op}}=\left[\begin{array}{c} \xi_{\text{op},1}\\ \xi_{\text{op},2,1}\\ \xi_{\text{op},2}\\ \xi_{\text{op},3,2}\\ \vdots \\ \xi_{\text{op},K} \end{array}\right],\;\epsilon =\left[\begin{array}{c} \epsilon_{1}\\ \epsilon_{2}\\ \vdots \\ \epsilon_{K} \end{array}\right], \end{array}$$

(90c)
$$\begin{array}{l} W=\mathrm{diag}\left(W_{1},W_{2,1},W_{2},\ldots ,W_{K,K-1},W_{K}\right), \end{array}$$

whereupon the quadratic approximation to the cost function can be written as

(91)
$${\left(\xi_{\text{op}}-H\epsilon \right)}^{T}W\left(\xi_{\text{op}}-H\epsilon \right).$$

Minimizing this with respect to ***ϵ*** results in a linear system of equations:

(92)
$$H^{T}WH\;\epsilon^{⋆}=H^{T}W\xi_{\text{op}}.$$

We solve for the optimal perturbations, ***ϵ***^⋆^, apply them to the initial guess according to (88), and iterate the process to convergence. This is again Gauss-Newton adapted to work for *SE*(3) and will converge locally in practice. We found a line search was needed for this problem to ensure smooth convergence (Nocedal and Wright, 1999).

##### B.5. Continuous-time trajectory estimation

To devise a local solver for (46), we begin with initial guesses, $\left\{T_{\text{op,k}},\varpi_{\text{op},k}\right\}$ , and perturb them according to

(93)
$$T_{k}=\text{cay}\left(\epsilon_{k}^{\wedge }\right)T_{\text{op,k}},\;\varpi =\varpi_{\text{op},k}+\eta_{k},$$

where $\epsilon_{k},\eta_{k}\in R^{6}$ are small unknown perturbations. This ensures that **T**_*k*_ ∈ *SE*(3) during the optimization process. A quadratic approximation to the cost function in (46) (in terms of the perturbations) is thus

(94)
$$\begin{array}{l} \overset{K}{\underset{k=1}{\sum }}{\left(\xi_{\text{op},k}-G_{k}x_{k}\right)}^{T}W_{k}\left(\xi_{\text{op},k}-G_{k}x_{k}\right)\\ +{\left({\overset{ˇ}{\varpi }}_{1}-\varpi_{\text{op},1}-E_{1}x_{1}\right)}^{T}Q_{1}^{-1}\left({\overset{ˇ}{\varpi }}_{1}-\varpi_{\text{op},1}-E_{1}x_{1}\right)\\ +\overset{K-1}{\underset{k=1}{\sum }}{\left(e_{\text{op},k+1,k}+F_{k}x_{k}-E_{k+1}x_{k+1}\right)}^{T}\;\\ \times \;Q_{k+1,k}^{-1}\left(e_{\text{op},k+1,k}+F_{k}x_{k}-E_{k+1}x_{k+1}\right), \end{array}$$

where

(95a)
$$\begin{array}{l} x_{k}=\left[\begin{array}{c} \epsilon_{k}\\ \eta_{k} \end{array}\right], \end{array}$$

(95b)
$$\begin{array}{l} e_{\text{op},k+1,k}=\left[\begin{array}{c} \left(t_{k+1}-t_{k}\right)\varpi_{\text{op},k}-\xi_{\text{op},k+1,k}\\ \varpi_{\text{op},k}-\varpi_{\text{op},k+1} \end{array}\right], \end{array}$$

(95c)
$$\begin{array}{l} G=\left[\begin{array}{cc} -J_{c}{\left(\xi_{\text{op},k}\right)}^{-1} & 0 \end{array}\right],\;E_{1}=\left[\begin{array}{cc} 0 & I \end{array}\right], \end{array}$$

(95d)
$$\begin{array}{l} F_{k}=\left[\begin{array}{cc} J_{c}{\left(\xi_{\text{op},k+1,k}\right)}^{-1}T_{\text{op},k+1,k} & \left(t_{k+1}-t_{k}\right)I\\ 0 & I \end{array}\right], \end{array}$$

(95e)
$$\begin{array}{l} E_{k+1}=\left[\begin{array}{cc} J_{c}{\left(\xi_{\text{op},k+1,k}\right)}^{-1} & 0\\ 0 & I \end{array}\right] \end{array}$$

with $\xi_{\text{op},k}={\text{cay}}^{-1}{\left(T_{\text{op},k}{\tilde{T}}_{k}^{-1}\right)}^{\vee }$ , $\xi_{\text{op},k+1,k}={\text{cay}}^{-1}{\left(T_{\text{op},k+1}T_{\text{op},k}^{-1}\right)}^{\vee }$ , and $T_{\text{op},k+1,k}=\text{Ad}\left(T_{\text{op},k+1}T_{\text{op},k}^{-1}\right)$ . We can tidy this up by defining the following^9^

(96a)
$$\begin{array}{l} F^{-1}=\left[\begin{array}{cccccc} E_{1} & 0 & 0 & \cdots & 0 & 0\\ -F_{1} & E_{2} & 0 & \cdots & 0 & 0\\ 0 & -F_{2} & E_{3} & \cdots & 0 & 0\\ \vdots & \vdots & \vdots & \ddots & \vdots & \vdots \\ 0 & 0 & 0 & \cdots & -F_{K-1} & E_{K} \end{array}\right], \end{array}$$

(96b)
$$\begin{array}{l} Q=\mathrm{diag}\left(Q_{1},Q_{2,1},\ldots ,Q_{K,K-1}\right), \end{array}$$

(96c)
$$\begin{array}{l} \;G=\mathrm{diag}\left(G_{1},G_{2},\ldots ,G_{K}\right), \end{array}$$

(96d)
$$\begin{array}{l} R=\mathrm{diag}\left(W_{1}^{-1},W_{2}^{-1},\ldots ,W_{K}^{-1}\right), \end{array}$$

(96e)
$$\begin{array}{l} e_{\text{op},v}=\left[\begin{array}{c} {\overset{ˇ}{\varpi }}_{1}-\varpi_{\text{op},1}\\ e_{\text{op},2,1}\\ \vdots \\ e_{\text{op},K,K-1} \end{array}\right],\;e_{\text{op},y}=\left[\begin{array}{c} \xi_{\text{op},1}\\ \xi_{\text{op},2}\\ \vdots \\ \xi_{\text{op},K} \end{array}\right], \end{array}$$

(96f)
$$\begin{array}{l} x=\left[\begin{array}{c} x_{1}\\ x_{2}\\ \vdots \\ x_{K} \end{array}\right], \end{array}$$

whereupon the cost function in (46) can be written as

(97)
$$\begin{array}{l} {\left(e_{\text{op},y}-Gx\right)}^{T}R^{-1}\left(e_{\text{op},y}-Gx\right)\\ +\;{\left(e_{\text{op},v}-F^{-1}x\right)}^{T}Q^{-1}\left(e_{\text{op},v}-F^{-1}x\right). \end{array}$$

Minimizing this with respect to **x** results in a linear system of equations:

(98)
$$\begin{array}{l} \left(G^{T}R^{-1}G+F^{-T}Q^{-1}F^{-1}\right)\;x^{⋆}\\ =\;G^{T}R^{-1}e_{\text{op},y}+F^{-T}Q^{-1}e_{\text{op},v}. \end{array}$$

We solve for the optimal perturbations, **x**^⋆^, unstack into $\epsilon_{k}^{⋆}$ and $\eta_{k}^{⋆}$ , then apply them to the initial guess according to (93), and iterate the process to convergence. This is again Gauss-Newton adapted to work for *SE*(3) and will converge locally in practice. We found a line search was needed for this problem to ensure smooth convergence (Nocedal and Wright, 1999). Note, if we do not have pose measurements at some of the estimation times, we can simply delete the appropriate rows/columns of **G**, **R**, and **e**_op,*y*_.
